# Inflammation-Responsive Hydrogels in Perioperative Pain and Wound Management: Design Strategies and Emerging Potential

**DOI:** 10.3390/gels11090691

**Published:** 2025-09-01

**Authors:** Young Eun Moon, Jin-Oh Jeong, Hoon Choi

**Affiliations:** 1 Department of Anesthesiology and Pain Medicine, Seoul St. Mary’s Hospital, College of Medicine, The Catholic University of Korea, Seoul 06591, Republic of Korea; momo0910@catholic.ac.kr; 2 Wake Forest Institute for Regenerative Medicine (WFIRM), Wake Forest School of Medicine, Winston-Salem, NC 27157, USA; jijeong@wakehealth.edu

**Keywords:** inflammation-responsive hydrogels, perioperative drug delivery, postoperative pain, wound healing, reactive oxygen species, pH-sensitive biomaterials, enzyme-responsive systems, surgical inflammation

## Abstract

Surgical procedures trigger dynamic inflammatory responses that influence postoperative pain, wound healing, and long-term outcomes. Conventional therapies rely on the systemic delivery of anti-inflammatory and analgesic agents, which often lack spatiotemporal precision and carry significant side effects. Inflammation-responsive hydrogels offer a promising alternative by enabling localized, stimulus-adaptive drug release aligned with the evolving biochemical milieu of surgical wounds. These smart biomaterials respond to endogenous triggers, such as reactive oxygen species, acidic pH, and proteolytic enzymes, allowing precise modulation of inflammation and tissue repair. This narrative review outlines the pathophysiological features of perioperative inflammation and the design principles of responsive hydrogel systems, including pH-, reactive oxygen species-, enzyme-sensitive, and multi-stimuli platforms. We evaluated the integration of key payloads, NSAIDs, corticosteroids, α_2_-adrenergic agonists, and biologics, highlighting their therapeutic synergy and translational relevance. Preclinical studies across soft tissue, orthopedic, thoracic, and abdominal models have demonstrated the efficacy of these systems in modulating immune responses, reducing pain, and enhancing regeneration. Despite these encouraging results, challenges remain, including trigger fidelity, surgical compatibility, and regulatory readiness. Future advances in biosensor integration, logic-based design, and artificial intelligence-guided formulation may accelerate clinical translation. Inflammation-responsive hydrogels represent a transformative strategy for precise perioperative care.

## 1. Introduction

Surgical procedures trigger a multifaceted cascade of physiological events collectively known as the perioperative inflammatory response. This process encompasses acute tissue damage, neurogenic inflammation, immune cell infiltration, and subsequent tissue remodeling, all of which contribute to postoperative pain and impaired wound healing [1]. While inflammation plays an essential role in tissue repair, its dysregulation can lead to excessive pain, delayed recovery, and complications such as fibrosis, chronic wounds, or infection [2,3,4].

Conventional approaches to managing perioperative inflammation and pain primarily rely on systemic pharmacological agents, including opioids, non-steroidal anti-inflammatory drugs (NSAIDs), and corticosteroids [5]. Although these agents are often effective, their use is limited by significant side effects, such as respiratory depression, gastrointestinal bleeding, immunosuppression, and interference with the natural healing process [6,7,8,9]. Furthermore, systemic administration lacks spatial and temporal precision, making it poorly suited to the localized and dynamic biochemical environments of surgical wounds [10,11].

Hydrogels have emerged as a promising class of biomaterials for localized drug delivery owing to their high water content, biocompatibility, mechanical tunability, and ability to conform to irregular tissue surfaces [12,13]. In particular, inflammation-responsive hydrogels have been engineered to dynamically react to pathological cues prevalent in inflamed tissues, such as acidic pH, elevated levels of reactive oxygen species (ROS), and increased protease activity [14,15,16]. By synchronizing drug release with the evolving wound microenvironment, these systems offer the potential to enhance therapeutic efficacy while minimizing off-target effects [17,18].

Despite these theoretical advantages, few inflammation-responsive hydrogel platforms have been specifically evaluated in perioperative pain or wound care models, particularly in clinically relevant surgical contexts. Most preclinical research to date has focused on chronic inflammation, traumatic injuries, or general regenerative applications [19,20]. This gap underscores the need to critically evaluate how inflammation-adaptive hydrogels can be effectively implemented in surgical settings, where precise timing, localized action, and immune modulation are essential.

This review explores the rationale, design strategies, and emerging therapeutic applications of inflammation-responsive hydrogels, with a focus on their role in perioperative pain management and wound healing. While previous reviews have examined hydrogel-based drug delivery in chronic wounds [21], cancer therapy [22], and regenerative medicine [23], perioperative applications remain underexplored as a distinct clinical domain. In contrast to these earlier efforts, our review integrates surgical pathophysiology, stimulus-specific hydrogel design, and translational considerations into a unified framework. Here, we synthesize current knowledge of perioperative inflammatory mechanisms with advances in pH-, ROS-, enzyme-, and multi-stimuli-responsive hydrogel platforms, critically assessing their performance across diverse surgical models and their readiness for clinical integration. We also highlight persistent translational barriers, including few precedents, stringent safety and biocompatibility requirements, and the absence of standardized regulatory evaluation protocols for responsiveness and degradation [24,25,26]. By bridging mechanistic insight with preclinical evidence and regulatory perspectives, this review offers a focused roadmap for developing next-generation biomarker-responsive hydrogel systems tailored to the unique temporal and spatial dynamics of surgical wounds.

In this review, the term “perioperative” is defined as the continuum encompassing the intraoperative period, the immediate postoperative phase (first 48–72 h), and the early recovery stage extending until wound closure and resolution of acute inflammation. This operational scope is selected to capture the dynamic inflammatory and nociceptive processes most relevant to the evaluation and optimization of hydrogel-based therapeutic interventions in surgical settings.

## 2. Pathophysiology and Design Implications of Perioperative Inflammation

Surgical trauma triggers a highly coordinated multifactorial inflammatory response involving molecular, cellular, and tissue-level events [1]. This response follows a tightly regulated temporal sequence, beginning with immediate immune activation and progressing through the resolution of inflammation and tissue regeneration [27]. Although this cascade is essential for wound healing, its dysregulation can lead to excessive pain, impaired repair, fibrosis, and other adverse outcomes [2].

A comprehensive understanding of the perioperative inflammatory landscape is essential to anticipate complications and guide the rational design of biomaterial-based interventions. Stimulus-responsive drug delivery systems must align their release kinetics with the dynamic, site-specific, and time-sensitive features of postoperative inflammation.

The following subsections examine the key molecular mediators, spatial and temporal dynamics, and endogenous biochemical triggers that define the perioperative wound microenvironment. These factors collectively inform the engineering of responsive hydrogels capable of synchronizing therapeutic actions with the evolving biology of surgical wounds.

### 2.1. Inflammatory Cascade Triggered by Surgical Trauma

Surgical injury initiates a complex and tightly regulated inflammatory cascade driven by damage-associated molecular patterns, pro-inflammatory cytokines, and innate immune cells. Upon tissue disruption, endogenous danger signals such as high-mobility group box 1, ATP, uric acid, and heat shock proteins are rapidly released from necrotic or stressed cells [28]. These molecules are recognized by pattern recognition receptors, including toll-like receptors and nucleotide-binding oligomerization domain-like receptors, which are expressed on both resident immune and parenchymal cells [29]. This recognition activates intracellular signaling pathways that trigger the release of key pro-inflammatory cytokines, including interleukin (IL)-1β, tumor necrosis factor-α (TNF-α), and IL-6 [30]. These cytokines mediate vasodilation, increase vascular permeability, and recruit circulating immune cells to the site of injury [31].

Neutrophils are among the first immune cells to infiltrate a wound and typically arrive within minutes to hours. They amplify the inflammatory response by releasing proteolytic enzymes, such as neutrophil elastase (NE) and matrix metalloproteinases (MMPs), as well as ROS, which contribute to tissue damage and inflammatory signaling [32]. Importantly, these early phase mediators represent viable biochemical triggers for stimulus-responsive drug delivery systems. As the response progresses, monocytes are recruited and differentiate into macrophages. Over time, these macrophages undergo phenotypic polarization from a pro-inflammatory M1 state to a pro-regenerative M2 phenotype, thereby facilitating tissue repair and remodeling [33]. This macrophage plasticity plays a pivotal role in determining whether the wound environment resolves toward regeneration or progresses to pathological fibrosis [34].

A detailed understanding of the temporal dynamics and functional roles of these inflammatory mediators is essential for designing responsive hydrogels. While early phase signals, such as ROS and neutrophil-derived enzymes, may enable rapid or burst-phase release, sustained MMP activity and cytokine gradients can guide prolonged or staged drug delivery. These mechanistic insights form the basis for engineering hydrogel platforms that can adapt to the evolving inflammatory landscape of surgical wounds.

### 2.2. Spatiotemporal Dynamics of the Inflammatory Response

Perioperative inflammatory response occurs through temporally and spatially dynamic processes. Broadly, it can be divided into an acute inflammatory phase (0–72 h) and a resolving or remodeling phase (>3 days), each defined by distinct cellular populations and molecular mediators. The acute phase is characterized by rapid neutrophil infiltration, elevated pro-inflammatory cytokine levels, oxidative stress, and tissue edema, all of which contribute to nociceptive sensitization and collateral tissue injury [35]. If unresolved, this phase may progress to chronic inflammation, marked by persistent macrophage activation, fibrotic remodeling, and impaired tissue regeneration [36].

Spatial heterogeneity further complicates the inflammatory landscapes. The severity and pattern of local inflammation can vary substantially across tissue compartments (e.g., muscle versus fascia) and even within a single wound. This variation is influenced by factors such as vascular density, oxygen gradients, and mechanical stress. Notably, surgical wounds often exhibit regions of localized hypoxia, which can aggravate inflammation via hypoxia-inducible factor-1α activation and lactic acid accumulation, ultimately lowering the extracellular pH to levels as low as 6.5 [37]. These microenvironmental features not only shape cellular behavior but also provide actionable biochemical cues for responsive drug delivery, particularly for pH-sensitive hydrogel systems.

From a therapeutic perspective, synchronizing drug release with the evolving inflammatory milieu is essential. Early phase interventions may benefit from rapid delivery of anti-inflammatory agents that target neutrophil-driven damage, whereas later stages may require controlled release of growth factors, cytokine inhibitors, or extracellular matrix (ECM) modulators to promote resolution and tissue regeneration [27]. To achieve this, inflammation-responsive hydrogels must be designed to accommodate both the temporal progression and spatial variability of surgical wounds, enabling phase-appropriate, site-specific, and adaptively modulated drug release.

### 2.3. Interplay Between Inflammation, Nociception, and Tissue Regeneration

The relationship between inflammation and nociception is a defining feature of the perioperative period. Pro-inflammatory cytokines such as TNF-α and IL-1β sensitize peripheral nociceptors by upregulating ion channels, including transient receptor potential vanilloid 1 and voltage-gated sodium channel Nav1.8. This molecular cascade promotes both peripheral and central sensitization, ultimately leading to hyperalgesia and prolonged postoperative pain [38]. Additionally, prostaglandin E2, which is synthesized via cyclooxygenase (COX)-2, further amplifies nociceptive signaling and contributes to the persistence of pain [39].

Immune cells involved in wound healing exert dual and sometimes opposing effects by concurrently modulating inflammation, nociception, and tissue repair. Macrophages are particularly important for maintaining this balance. Classically activated M1 macrophages secrete ROS and pro-inflammatory cytokines that exacerbate pain signaling, whereas alternatively activated M2 macrophages promote angiogenesis, ECM remodeling, and regenerative healing [40]. Thus, the phenotypic plasticity of macrophages reflects a broader biological tension: reducing inflammation may alleviate pain but could also hinder tissue repair, whereas permitting inflammatory signaling may support regeneration at the cost of sustained nociception.

Beyond serving as passive drug depots, inflammation-responsive hydrogels can actively modulate these wound-healing cascades through intrinsic biophysical and biochemical cues. Matrix stiffness, degradability, and ligand presentation (e.g., RGD, GFOGER) can bias macrophage polarization toward pro-regenerative phenotypes, modulate fibroblast activity, and support angiogenesis and organized collagen deposition [41]. Redox-active or ROS-scavenging backbones reduce oxidative stress and neutrophil-mediated injury [42], while ECM-mimetic or electrostatic chemistries can sequester proteases and cytokines to mitigate edema [43,44]. These host–material interactions operate in parallel with payload release and are increasingly recognized as co-determinants of analgesic efficacy and regenerative outcomes in perioperative wounds.

Addressing this complex interplay requires delivery systems that can adapt to the inflammatory phase, modulating therapeutic responses in a temporally and spatially precise manner. Inflammation-responsive hydrogels offer such capability, aligning analgesic effects with regenerative processes and thereby improving surgical wound care outcomes.

### 2.4. Biomarkers as Triggers for Responsive Drug Release

The inflammatory microenvironment of surgical wounds contains a diverse array of biochemical signals that serve as precise and biologically relevant triggers for on-demand drug delivery. These cues are characterized by their spatial and temporal specificity, and have been successfully utilized in the design of inflammation-responsive hydrogel systems. Key categories include the following:

- pH shifts: Local acidosis, typically driven by hypoxia and lactic acid accumulation, can lower the extracellular pH to approximately 6.5 during the acute inflammatory phase [45]. Hydrogels incorporating acid-labile linkers, such as orthoesters, imines, and acetals, can be engineered to swell, degrade, or release therapeutic agents selectively under mildly acidic conditions, thereby enabling rapid and localized drug delivery [46].
- ROS: Neutrophil-derived ROS, including hydrogen peroxide and superoxide, are elevated in inflamed and ischemic tissues following surgical injury [47]. Hydrogels containing ROS-sensitive moieties such as thioketal or boronic ester groups can undergo cleavage or structural transitions in response to oxidative stress, allowing either burst or sustained drug release [19].
- Proteolytic enzymes: Proteases such as MMP-2, MMP-9, and NE are commonly upregulated in inflamed surgical wounds [32]. Peptide-based crosslinkers designed to be cleaved by these enzymes enable site-specific degradation of hydrogels, facilitating targeted therapeutic release at protease-rich sites [48].
- Cytokine gradients: Although more challenging to directly exploit, inflammatory cytokines such as IL-1β and TNF-α can inform the development of feedback-controlled delivery systems. Aptamers are short, single-stranded nucleic acids that bind specific molecular targets with high affinity and selectivity. When incorporated into a hydrogel matrix, these aptamer-functionalized systems can undergo conformational or charge-based changes upon binding their target cytokine, triggering structural transitions or payload release. Alternatively, nanoparticle-integrated hydrogel matrices can be engineered to sense cytokine concentrations through particle surface chemistry or embedded biosensing elements, enabling dynamic modulation of drug release in response to inflammatory status [49,50].

Table 1 summarizes the relationship between key inflammatory phases, representative biochemical triggers, and design considerations for responsive hydrogel systems in the perioperative context.

These molecular cues are not only pathophysiologically relevant but also structured in a temporally and spatially defined manner, which is essential for achieving precise inflammation-adaptive drug delivery [51]. By aligning the therapeutic release kinetics with endogenous inflammatory signals, responsive hydrogel systems can enhance local efficacy, reduce systemic exposure, and adapt more effectively to the evolving biochemical landscape of surgical wounds.

Although most stimulus-responsive hydrogel platforms have been developed for chronic or nonsurgical inflammatory conditions, the perioperative wound environment offers a uniquely well-defined and temporally constrained biochemical profile. Transient pH fluctuations, oxidative bursts, protease activity, and cytokine surges provide a robust and clinically actionable framework for responsive material design.

In summary, the biological complexity of perioperative inflammation represents not only a therapeutic challenge but also a valuable engineering opportunity. Smart hydrogel systems that harness endogenous inflammatory signals offer a compelling path toward adaptive, targeted, and clinically translatable solutions for perioperative pain management and wound healing.

## 3. Design Principles of Inflammation-Responsive Hydrogels

**Figure 1:**
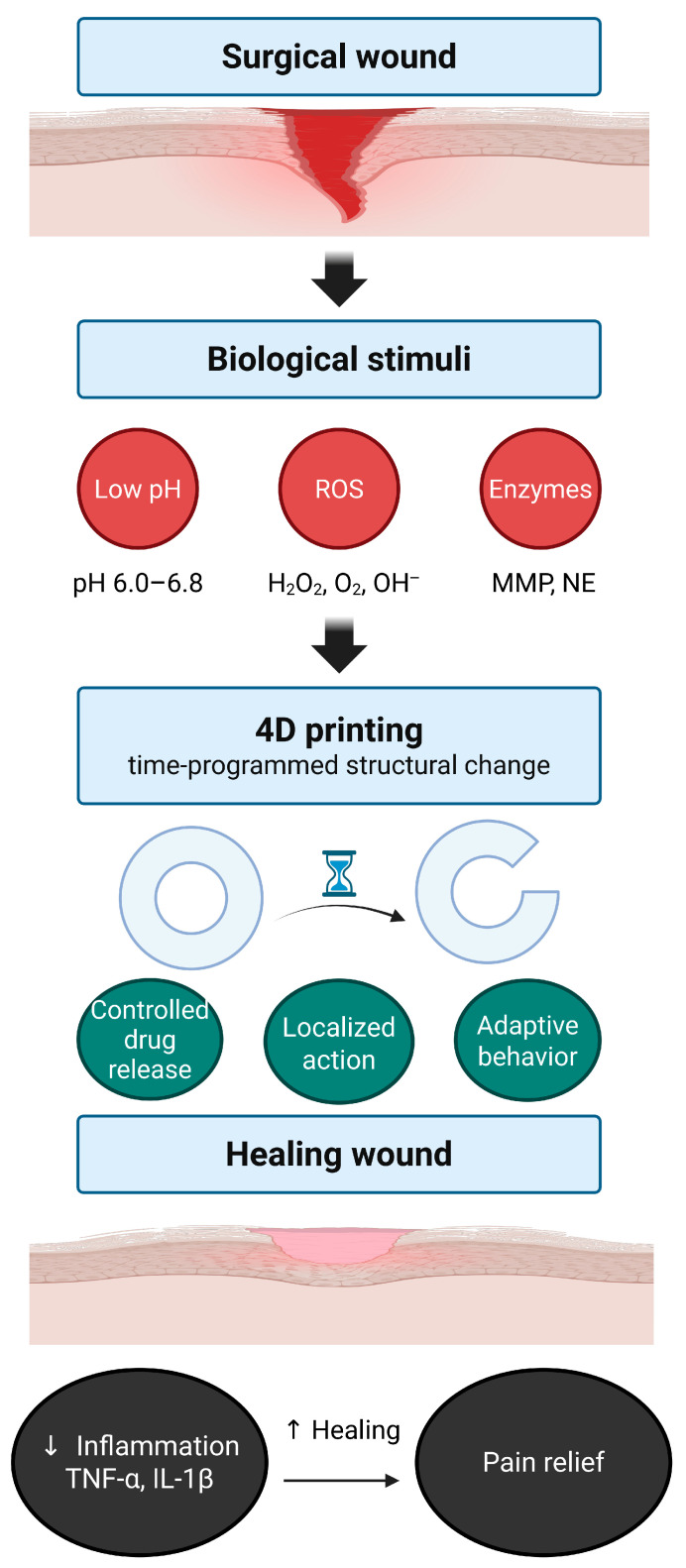
Conceptual schematic linking 4D printing to inflammation-responsive hydrogels. Surgical wounds generate biological stimuli such as local acidosis (pH 6.0–6.8), reactive oxygen species (H_2_O_2_, O_2_^−^, OH^−^), and proteolytic enzymes (MMP, NE). These cues trigger time-programmed structural changes in hydrogel systems, analogous to the concept of 4D printing. As a result, hydrogels undergo material responses (swelling, degradation, bond cleavage) leading to functional outputs such as controlled drug release, localized action, and adaptive behavior. These mechanisms promote reduced inflammation (↓TNF-α, IL-1β), accelerated healing, and pain relief in perioperative settings.

The broader concept of stimuli-responsive materials can be traced back to the field of 4D printing. The term “4D printing” was first introduced in 2013, describing three-dimensional constructs that are programmed to change shape, properties, or function over time in response to predefined environmental cues [52]. This paradigm laid the groundwork for materials whose performance could be dynamically modulated by stimuli such as temperature, pH, light, or moisture [53]. Importantly, this framework provided the conceptual foundation for biomedical engineering, where responsiveness could be tailored not only to physical but also to biochemical cues within tissue environments. Building on this foundation, polymeric hydrogel systems were developed to alter their structure or release profiles in response to wound-associated signals such as acidosis, oxidative stress, and elevated protease activity (Figure 1).

Successful translation of perioperative pathophysiology into therapeutic outcomes requires the development of material systems capable of detecting and responding to dynamic biological signals. As described in the previous section, surgical wounds generate a temporally ordered sequence of biochemical cues, including local acidosis, oxidative stress, and elevated protease activity, which vary spatially and temporally. These pathophysiological features create a favorable context for inflammation-responsive hydrogels to deliver therapeutics in a localized, adaptive, and phase-specific manner [18].

To harness these cues, hydrogel systems are engineered with chemical motifs that undergo structural or physicochemical changes, such as swelling, degradation, or bond cleavage, in response to defined stimuli. Most existing platforms utilize acid-labile linkages, ROS-sensitive groups, or enzyme-cleavable crosslinkers to enable site- and condition-specific drug release. More advanced designs incorporate multi-stimuli responsiveness or logic-gated mechanisms to enhance spatial precision, reduce off-target activation, and accommodate the complexity of inflamed surgical environments [12].

Although inflammation-responsive hydrogels have been widely studied in models of chronic inflammation, trauma, and regenerative medicine, their application in perioperative care remains relatively underexplored. Nevertheless, the predictable and transient nature of postoperative inflammation presents a unique opportunity for temporally coordinated and spatially selective drug delivery.

The following subsections classify inflammation-responsive hydrogels into four main categories: pH-responsive, ROS-responsive, enzyme-responsive, and multi-stimuli or feedback-controlled systems. For each type, we describe the underlying chemical design strategies, stimulus-response mechanisms, and potential applications in perioperative pain management and wound healing.

### 3.1. pH-Responsive Hydrogels

One of the most well-characterized features of inflamed surgical tissues is localized extracellular acidosis. Following trauma, hypoxia and elevated glycolytic metabolism lead to lactic acid accumulation, reducing extracellular pH to as low as 6.0–6.8 during the acute postoperative phase [54]. Transient acidosis provides a reliable endogenous trigger for pH-responsive hydrogel systems.

These hydrogels are designed to undergo structural transformations, such as swelling, degradation, or sol–gel transitions, when exposed to mildly acidic environments. The triggering mechanism generally involves protonation of ionizable groups or acid-catalyzed hydrolysis of labile covalent bonds, which destabilizes the polymer network and alters mesh size or hydrophilicity, thereby accelerating drug diffusion. In acid-labile linkages, the drop in pH facilitates cleavage of susceptible bonds (e.g., orthoesters, ketals), while in polyelectrolyte systems, protonation reduces electrostatic repulsion and drives network contraction or dissolution. These responses are typically mediated by acid-labile chemical linkages or ionizable functional groups embedded in a polymer matrix [14]. Common strategies include the following:

- Orthoesters, acetals, and ketals: These linkages undergo hydrolytic cleavage under acidic conditions, leading to network disassembly and accelerated drug release [55,56].
- Schiff base (imine) bonds: Formed through aldehyde–amine condensation, these bonds are relatively stable at physiological pH, but hydrolyze rapidly under acidic conditions [57].
- Poly(acrylic acid) (PAA) derivatives: These polymers undergo pH-dependent ionization of the carboxyl groups, enabling reversible swelling through protonation and deprotonation [58].

The pH responsiveness of these systems can be finely tuned by adjusting the polymer composition, crosslinking density, and pKa of the functional groups to ensure activation at pH levels below 6.8. This helps avoid premature or off-target drug release in non-inflamed tissues [59].

Several design examples illustrate the versatility and translational potential of this strategy:

- A mesoporous glass–hydroxyapatite scaffold incorporating orthoester linkers achieved pH-triggered levofloxacin release under acidic conditions (pH 5.5–6.7), enhancing bacterial clearance while preserving osteoblast viability [60]. Although developed for bone infections, this platform can be adapted for localized anti-inflammatory delivery in acidified surgical wounds.
- A self-healing polyethylene glycol (PEG)-based hydrogel, crosslinked via imine bonds between aldehyde- and amine-functionalized poly(ether urethane)s, remained stable at pH 7.4, but was hydrolyzed in acidic environments. It supported sustained drug release for up to 17 days and was injectable through a 21 G needle, suggesting promise for minimally invasive perioperative applications in spatially and temporally confined inflammation [61].
- A semi-interpenetrating κ-carrageenan–PAA hydrogel, originally developed for gastrointestinal diclofenac delivery, exhibited pH-dependent diffusion (~80% at pH 7.4 vs. ~40% at pH 1.2) [62]. This behavior may be repurposed to match drug availability with wound pH normalization during postoperative recovery.

pH-responsive hydrogels leverage a wide range of acid-sensitive chemistries, including covalent bond cleavage and ionic swelling, to achieve controlled drug release in acidic wound environments. These systems function autonomously, require no external input, and have shown therapeutic efficacy in multiple models of inflammation and infection. Although their application in surgical settings is still emerging, the predictable presence of acidosis in postoperative wounds highlights the potential of pH-sensitive platforms for precise and adaptive drug delivery during perioperative care.

### 3.2. ROS-Responsive Hydrogels

Oxidative stress is a hallmark of acute postoperative inflammation. Following surgical trauma, activated neutrophils and macrophages produce elevated levels of ROS, including hydrogen peroxide, superoxide, and hydroxyl radicals [63]. Although physiological levels of ROS contribute to host defense and cellular signaling, excessive ROS accumulation leads to collateral tissue damage, prolonged inflammation, and impaired wound healing [64].

ROS-responsive hydrogels are engineered to leverage this oxidative microenvironment by incorporating chemical moieties that undergo bond cleavage, oxidation-induced polarity changes, or crosslink density modulation in the presence of ROS. The triggering mechanism typically involves oxidation of sulfur-, boron-, or selenium-containing groups, which disrupts covalent linkages or alters hydrophilicity, thereby increasing mesh size and facilitating drug diffusion. In some cases, these chemical transformations simultaneously generate antioxidant byproducts, offering intrinsic ROS-scavenging activity alongside drug release [15]. Three primary design strategies are commonly employed:

- Thioketal linkages (–S–C–S–): These linkages are selectively cleaved in the presence of ROS, resulting in hydrogel disassembly and controlled drug release [65].
- Boronic esters or acids: These groups react specifically with hydrogen peroxide to form phenols and boric acid, triggering gel degradation or drug release [66].
- Selenium-containing moieties (e.g., diselenide bonds): Upon oxidation, these are converted to selenoxide derivatives, modulating the crosslink density and often imparting intrinsic antioxidant activity [67].

These mechanisms allow for tunable drug release kinetics, and some platforms offer the added benefit of ROS scavenging. The degradation threshold and response rate can be adjusted to match the oxidative burden in inflamed surgical tissues [15].

Representative systems illustrate the versatility and perioperative relevance of this approach:

- A PEG hydrogel crosslinked with thioketal linkages was developed for the ROS-responsive delivery of epidermal growth factor. Upon ROS exposure, thioketal cleavage accelerated growth factor release, leading to enhanced angiogenesis, reduced oxidative stress, and improved re-epithelialization in a full-thickness wound model [68]. Compared with non-responsive controls, this system significantly improved wound closure, demonstrating direct applicability to surgical incisions with oxidative injury.
- A dual-responsive gelatin–poly(vinyl alcohol) (PVA) hydrogel incorporating boronic ester crosslinks and pH-sensitive micelles enabled the sequential release of vancomycin–silver nanoclusters and nimesulide. ROS triggered rapid antimicrobial delivery, while acidic pH sustained anti-inflammatory release. In a diabetic infected wound model, this system outperformed commercial dressings and single-drug controls in bacterial clearance, cytokine suppression, and hemostasis [69]. Although developed for chronic wounds, its dual-triggered logic and infection-modulating capacity are relevant in perioperative settings, particularly at contaminated or high-risk surgical sites.
- A selenium-based polyurethane hydrogel incorporating diselenide crosslinkers responded to oxidative stress by forming selenoxide, softening the matrix, and delivering antioxidant effects. In a rat myocardial infarction model, this system reduced fibrosis, suppressed inflammatory cytokines, and improved cardiac function relative to non-selenium controls [70]. Its redox-adaptive behavior is especially pertinent to cardiovascular and thoracic surgeries that involve localized oxidative injury.

ROS-responsive hydrogels offer a compelling strategy for inflammation-adaptive drug delivery, particularly during the early postoperative period when oxidative stress peaks. By integrating therapeutic release with intrinsic ROS-scavenging capabilities, these systems provide the dual benefits of mitigating tissue damage and modulating immune responses. Moving forward, emphasis should be placed on validating these platforms in surgical models, with a focus on achieving precise temporal alignment between oxidative cues and drug release profiles.

### 3.3. Enzyme-Responsive Hydrogels

Proteolytic enzymes are key mediators of inflammation, ECM remodeling, and immune activation following surgical trauma. MMPs, particularly MMP-2 and MMP-9, play essential roles in ECM degradation, angiogenesis, and wound resolution [71]. NE, released by activated neutrophils, contributes to antimicrobial defense, but can exacerbate tissue injury when dysregulated [32]. The localized and temporally regulated expression of these enzymes makes them attractive targets for stimulus-responsive hydrogel systems.

Enzyme-responsive hydrogels are engineered by incorporating protease-cleavable peptide sequences into the polymer backbone, crosslinks, or pendant chains. Upon recognition and hydrolysis by the target protease, these sequences undergo specific peptide bond cleavage, disrupting the network architecture and increasing mesh size. This structural change facilitates drug diffusion or complete matrix degradation, depending on crosslink density and polymer hydrophilicity. Common biochemical motifs include GPLGIAGQ or CVPLSLYSG for MMP-2/9 and AAPV for NE, which are selectively cleaved at physiological temperature and pH. These cleavable linkers can be integrated into bulk hydrogels, microneedles, or nanoparticle–hydrogel hybrids, enabling precise spatiotemporal drug release in protease-rich postoperative environments [48]. Common design strategies include the following:

- MMP-cleavable linkers (e.g., GPLGIAGQ, CVPLSLYSG) integrated into PEG-based matrices to enable enzyme-responsive degradation and drug release [72].
- NE-sensitive motifs (e.g., AAPV) for targeted activation in neutrophil-rich tissues, especially during early postoperative inflammation [73].
- Microneedle and nanoparticle–hydrogel hybrids that combine physical precision with enzyme-triggered delivery, supporting minimally invasive surgical applications [74].

These strategies enable spatiotemporally regulated drug delivery aligned with dynamic protease activity in surgical wounds.

Several examples have highlighted the translational potential of this approach:

- A tetra-PEG hydrogel crosslinked with GPLGIAGQ peptides was used for the MMP-2-responsive delivery of phosphatidylserine. This system promoted M2 macrophage polarization, suppressed IL-1β and TNF-α expression, and enhanced bone regeneration in a rat calvarial defect model. Compared with drug-free and non-responsive controls, the hydrogel showed superior immunomodulatory effects, supporting its relevance for surgical tissue repair [75].
- A PEG hydrogel incorporating CVPLSLYSG linkers was engineered for MMP-2/9-responsive delivery of docetaxel-loaded poly(lactic-co-glycolic acid) (PLGA) nanoparticles. Upon enzyme exposure, the matrix degraded, releasing nanoparticles that achieved over five-fold higher cytotoxicity against glioma cells compared to free docetaxel, and over 20-fold compared to non-degradable controls [76]. Although developed for post-resection chemotherapy, this design underscores the spatial precision achievable in surgical oncology.
- An NE-responsive RADA16-I hydrogel functionalized with AAPV-cleavable sequences and regenerative peptides (GHK, KGHK, and RDKVYR) released bioactive cues upon NE exposure. In a murine full-thickness wound model, the system accelerated re-epithelialization, enhanced fibroblast activity, and promoted collagen deposition, demonstrating its efficacy in protease-rich wound environments [77].
- A microneedle patch composed of gelatin methacryloyl (GelMA) and ε-poly-L-lysine loaded with curcumin nanoparticles was developed for MMP-triggered degradation. In infected wound models, the patch enhanced closure, suppressed TNF-α, and upregulated IL-10 and vascular endothelial growth factor (VEGF) expression [78].

Enzyme-responsive hydrogels represent a flexible and targeted approach to perioperative drug delivery, capitalizing on the temporally and spatially restricted activities of pathological proteases. Their selective responsiveness enables context-specific release of immunomodulators, antimicrobial agents, or regenerative factors. Future developments should prioritize multi-enzyme integration and validation in surgical models that reflect real-world inflammatory complexity.

### 3.4. Multi-Stimuli and Feedback-Controlled Systems

The postoperative wound environment presents a complex and evolving landscape, characterized by overlapping inflammatory signals, such as oxidative stress, local acidosis, and protease activity. Although hydrogels responsive to a single stimulus can offer targeted drug delivery, their therapeutic precision may be suboptimal under such multifactorial conditions. To improve responsiveness and adaptability, next-generation hydrogel platforms have been developed to integrate multiple endogenous triggers and incorporate autonomous control systems. These strategies aim to enable context-sensitive, phase-appropriate drug release tailored to the dynamic physiology of surgical wounds [79].

Multi-stimuli-responsive hydrogels have been engineered to respond to two or more pathological cues simultaneously, thereby enhancing delivery specificity in heterogeneous and temporally variable inflammatory environments. Mechanistically, these systems integrate distinct chemical motifs, such as acid-labile linkers and ROS-sensitive groups, so that protonation and oxidation act in parallel or synergistically to weaken crosslinks, expand mesh size, and accelerate drug diffusion [80]. One example is an octacalcium phosphate (OCP)-laden alginate hydrogel, in which OCP particles are uniformly dispersed within an ionically crosslinked alginate matrix. This platform not only offers osteoconductive properties but also exhibits improved stability under simulated inflammatory conditions, including acidic pH and elevated ROS levels, by reducing hydrogel degradation and maintaining structural integrity over prolonged exposure [81]. Such characteristics are particularly advantageous in surgical environments where biochemical and mechanical challenges coexist, suggesting potential for long-term, inflammation-resilient therapeutic delivery.

Building on the multi-stimuli concept, Boolean logic–based hydrogels apply combinatorial rules to further refine responsiveness, with AND gates requiring multiple stimuli simultaneously and OR gates triggered by any one of several stimuli [82]. A representative example is an OR-gated platform composed of ROS-sensitive GelMA and carboxyphenylboronic acid (CPBA), combined with a pH-cleavable oxidized chondroitin sulfate (CS) matrix encapsulating curcumin-loaded α-lipoic acid–grafted chitosan micelles. This system released its payload under either oxidative or acidic conditions and achieved over 95% wound closure by day 14 in infected wound models. Compared to micelle-only, hydrogel-only, and phosphate-buffered saline controls, the treatment significantly enhanced collagen deposition, angiogenesis, and M2 macrophage polarization [83]. Although developed for dermal applications, the system’s dual responsiveness and inflammation-adaptive logic suggest a strong potential for managing contaminated or high-risk surgical wounds.

Feedback-controlled hydrogel systems, in the strict sense, actively regulate drug release through real-time monitoring of endogenous biochemical signals, thereby forming a closed loop between sensing and therapeutic output. This approach differs fundamentally from predefined stimulus-responsive designs, which passively release payloads upon encountering a pathological cue without continuous feedback. True feedback platforms typically integrate molecular, optical, or electrochemical biosensors within the hydrogel matrix to detect fluctuations in clinically relevant biomarkers and trigger therapeutic release accordingly [84]. One notable strategy involves aptamer-functionalized hydrogel matrices designed for biosensing and targeted delivery. In a recent study, a photopatternable hydrogel composed of norbornene-functionalized PVA embedded with thiolated DNA aptamers that recognize serotonin and dopamine was developed. Two-photon polymerization enabled microscale patterning (~2.17 µm resolution), maintaining mechanical stability as confirmed by fluorescence recovery after photobleaching and rheological analysis [85]. While initially intended for neurochemical sensing, the modular aptamer framework could be adapted to detect inflammatory biomarkers, such as IL-6, TNF-α, or C-reactive protein, potentially enabling closed-loop perioperative wound monitoring and therapy.

Redox-switchable hydrogels offer yet another strategy, particularly for pulsatile or repeated drug delivery in oxidative surgical environments [86]. A dual-crosslinked poly(N-isopropylacrylamide) system incorporating both N,N′-methylenebisacrylamide and redox-sensitive N,N′-bis(acryloyl)cystamine crosslinkers enabled reversible swelling and protein release in response to redox cycling. Under reducing conditions, disulfide bonds were cleaved to allow hydrogel expansion and therapeutic release. Under oxidative conditions, the network re-formed, allowing mechanical recovery and reloading with over 83% efficiency [87]. This system remained functional over multiple on-off cycles under flow conditions and may serve as a depot-like dosing platform during postoperative recovery, particularly where fluctuating oxidative stress is anticipated.

Together, these systems mark a significant step toward autonomous inflammation-responsive therapy in perioperative care. By integrating multiple stimuli or embedding biosensing modules, real-time spatially localized control of drug release in complex wound environments can be achieved. Future research should focus on expanding their responsiveness to clinically relevant inflammatory targets, coupling them with wearable biosensors, and validating their efficacy in surgical models to support their incorporation into personalized recovery protocols [88].

Although these platforms exploit physiologically relevant cues, the magnitude and temporal profile of pH, ROS, and protease activity are highly context dependent across patients and surgical procedures. Surgical and wound milieus can exhibit pronounced spatial pH gradients and temporal acidity shifts driven by hypoxia and lactate metabolism, varying with tissue type and perfusion status [45,54]. Similarly, ROS levels fluctuate with ischemia–reperfusion events, immune cell dynamics, and baseline metabolic activity; even non-inflammatory ROS may prematurely cleave ROS-labile linkers such as boronate esters and thioketals if selectivity is insufficient [63,65]. Protease activity (e.g., MMP-2/9, NE) likewise shows patient- and lesion-specific variability and changes markedly between healing and fibrotic phases, complicating the prediction of activation kinetics for enzyme-cleavable networks [27,71,73].

This heterogeneity can lead to failure modes including false positives (premature release in mildly perturbed tissue), false negatives (under-release in hypo-inflammatory niches), and payload depletion before the critical therapeutic window. To mitigate these risks and discriminate between physiological and pathological levels of these stimuli, hydrogel systems can be engineered with (i) calibrated activation thresholds and buffer capacity tuning to confine responsiveness within pathological ranges [89], (ii) logic-gated multi-stimuli designs (AND/OR) that require concurrent pathological signals to trigger release [90], and (iii) feedback-guided or adaptively crosslinked networks capable of adjusting release kinetics to evolving wound cues in situ [91]. From a translational standpoint, achieving high trigger fidelity and context specificity remains a central challenge, and should be validated against clinically relevant ranges and benchmarks before surgical use [12].

**Figure 2:**
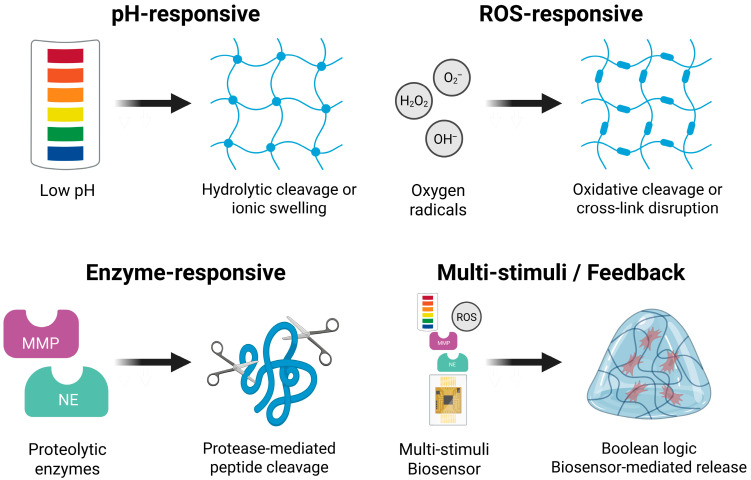
Schematic overview of the main classes of inflammation-responsive hydrogels. Distinct categories include pH-responsive systems activated by local acidosis (pH 6.0–6.8), ROS-responsive systems triggered by oxidative radicals (H_2_O_2_, O_2_^−^, OH^−^), enzyme-responsive systems cleaved by proteolytic enzymes (MMP, NE), and multi-stimuli or feedback-controlled systems that integrate logic gates or biosensors. Each class exhibits characteristic response mechanisms such as hydrolytic bond cleavage, oxidative cross-link disruption, protease-mediated peptide degradation, or Boolean logic–based release, enabling context-specific therapeutic delivery.

By incorporating molecular motifs that respond selectively to pH fluctuations, oxidative stress, or proteolytic activity, and by integrating multi-stimuli or feedback-controlled strategies, inflammation-responsive hydrogels can dynamically adapt to evolving wound environments. Their modular design offers a promising framework for tailoring drug release profiles to distinct phases of perioperative healing and inflammation. A concise comparison of these hydrogel classes is presented in Table 2, while Figure 2 provides a schematic overview of their representative triggers, response mechanisms, and design strategies relevant to perioperative applications.

## 4. Anti-Inflammatory and Analgesic Payloads for Hydrogel Delivery

Effective management of perioperative inflammation and pain is essential for optimal surgical recovery. Beyond alleviating discomfort, timely modulation of these responses facilitates tissue regeneration, prevents complications, and reduces the risk of chronic postsurgical pain. However, the conventional systemic administration of anti-inflammatory and analgesic drugs often suffers from poor tissue selectivity, narrow therapeutic windows, and significant systemic side effects, ultimately limiting their clinical efficacy [92].

Inflammation-responsive hydrogels offer a compelling alternative by enabling the localized, sustained, and stimulus-adaptive delivery of therapeutic agents. These systems can encapsulate diverse payloads, including NSAIDs, corticosteroids, α_2_-adrenergic agonists, and biologics with immunomodulatory or regenerative activity. Through responsiveness to pH, ROS, or proteolytic cues, drug release can be temporally aligned with the evolving wound microenvironment [93]. Concurrently, tuning hydrogel properties such as porosity, charge, and hydrophilicity can further optimize release kinetics, drug stability, and local bioavailability [94].

The following subsections examine the key classes of therapeutic payloads integrated into inflammation-responsive hydrogel systems with relevance to perioperative care. We first review NSAIDs and corticosteroids for inflammatory cascade suppression, followed by α_2_-adrenergic agonists that offer both analgesic and immunomodulatory effects. Next, we explore biologics aimed at wound healing and immune regulation, and conclude with dual-payload strategies designed to synergize therapeutic effects under dynamic inflammatory conditions.

### 4.1. Non-Steroidal Anti-Inflammatory Drugs (NSAIDs)

NSAIDs are widely used in perioperative care owing to their analgesic and anti-inflammatory properties. They reduce both pain and tissue inflammation by inhibiting COX enzymes and suppressing prostaglandin synthesis. However, systemic administration, whether oral, intravenous, or intramuscular, frequently results in adverse effects, such as gastrointestinal irritation, renal dysfunction, and cardiovascular risk, especially in elderly patients or those with comorbidities [6,95].

Hydrogel-based NSAID delivery offers a localized and sustained alternative that may mitigate these systemic toxicities [96]. In particular, inflammation-responsive platforms can further enhance therapeutic precision by synchronizing drug release with local cues, such as pH, oxidative stress, or protease activity [18].

A notable example is a dual pH- and ROS-responsive hydrogel composed of PAA-co-N-acryloxysuccinimide), carboxymethyl chitosan (CMCS), poly(hexamethylene guanidine), and manganese dioxide nanoparticles, which facilitated diclofenac release under inflammatory conditions. In an infected wound model, the system achieved 87.6% wound closure by day 6, promoted angiogenesis, and suppressed TNF-α and IL-1β compared to component-only and drug-free controls, supporting its potential for contaminated surgical sites [97].

Another study utilized a Carbopol EZ-3 hydrogel containing ketoprofen, menthol, and surfactants, which enhanced dermal drug penetration and reduced carrageenan-induced paw edema in rats. Although this system lacked inflammation-responsiveness, it outperformed commercial diclofenac cream in terms of local anti-inflammatory efficacy [98]. Its formulation can be adapted into a responsive hydrogel tailored for perioperative applications.

In a separate approach, a thermosensitive hydrogel composed of hyaluronic acid (HA) and poloxamer 407 was used to deliver ketoprofen-loaded transethosomes for intra-articular administration. In a rat osteoarthritis model, the system attenuated pro-inflammatory cytokines (TNF-α, IL-1β, IL-6, and IL-22) and preserved the cartilage structure, indicating its relevance to postoperative inflammation in orthopedic or arthroscopic settings [99].

An MMP-13–responsive microsphere system utilizing HA methacrylate and cationic liposomes enabled enzyme-triggered celecoxib release. In vivo, it significantly reduced TNF-α and MMP-13 expression and improved joint histology relative to non-responsive and drug-free controls [100]. This underscores the value of protease-adaptive delivery for managing COX-2–mediated inflammation during tissue remodeling.

Collectively, these examples demonstrate that inflammation-responsive hydrogels can improve NSAID pharmacokinetics, enhance the local anti-inflammatory efficacy, and reduce systemic toxicity. Although clinical translation is still in its early stages, these systems represent a promising addition to multimodal perioperative analgesic strategies.

### 4.2. Corticosteroids

Corticosteroids are potent anti-inflammatory agents that are frequently employed in surgical settings to mitigate acute inflammation, reduce edema, and prevent fibrotic remodeling. Through the activation of glucocorticoid receptors and inhibition of transcription factors such as NF-κB and AP-1, they suppress pro-inflammatory cytokine production, attenuate leukocyte infiltration, and stabilize vascular permeability [101]. Dexamethasone and triamcinolone are particularly favored for perioperative applications owing to their high potency and long duration of action [102].

Nevertheless, systemic or repeated local administration of corticosteroids is associated with adverse effects, including immunosuppression, delayed wound healing, and soft tissue atrophy [103]. To minimize these risks while preserving therapeutic efficacy, hydrogel-based delivery platforms have been developed to localize and prolong corticosteroid release. Inflammation-responsive hydrogels, in particular, enable stimulus-adaptive release in response to cues such as oxidative stress, acidic pH, or elevated protease activity [104,105,106].

One example is a thermosensitive PEG–poly(ε-caprolactone)–PEG hydrogel loaded with dexamethasone-encapsulated micelles. This injectable formulation gelled in situ and showed sustained release for over 14 days. In a rat peritoneal adhesion model, it significantly reduced adhesion scores, suppressed inflammatory cell infiltration, and promoted mesothelial regeneration, outperforming free micelles and blank gels [107]. Although not responsive, its temporal alignment with postoperative inflammation suggests its adaptability to responsive designs.

In the spinal surgery context, a PEG-based DexaPatch was developed using 4-arm PEG–maleimide, poloxamer-derived dithiol crosslinkers, and PLGA microparticles for local dexamethasone delivery. The rehydrated patch demonstrated mechanical robustness and minimal swelling, making it suitable for use in confined surgical spaces. In a rabbit cervical discectomy model, it reduced prevertebral edema and fibrotic changes [108]. Although the platform lacked direct responsiveness, its intraoperative applicability supports future integration with inflammation-triggered release mechanisms.

Triamcinolone acetonide has also been incorporated into advanced composite hydrogels. A Janus Tough Adhesive system composed of alginate–polyacrylamide and a chitosan surface layer was loaded with triamcinolone microcrystals. In a rat Achilles tendon repair model, the hydrogel reduced scarring, inhibited neovascularization, and promoted M2 macrophage polarization [109]. Although not formally stimulus-responsive, the chitosan layer enhanced adhesion in acidic environments, suggesting its potential benefits in inflamed surgical wounds.

A more explicitly responsive system incorporated triamcinolone into a collagenase-degradable gelatin sheet crosslinked by dehydrothermal treatment. In a murine dorsal wound model, this hydrogel released the drug in response to elevated collagenase activity, suppressed myofibroblast infiltration, and reduced fibrosis compared to intradermal steroid injection or blank controls. While epithelialization was modestly delayed, the system promoted more uniform remodeling, indicating its potential utility in soft tissue surgery [110].

Altogether, these examples underscore the potential of corticosteroid-loaded hydrogels to provide localized, sustained, and stimulus-adaptive anti-inflammatory therapy in perioperative settings. Platforms that incorporate enzyme-responsive or environmentally adaptive features may enhance therapeutic precision while minimizing systemic side effects. Corticosteroids may also be integrated into dual-function hydrogel systems with NSAIDs, anesthetics, or regenerative agents to support the coordinated control of inflammation, pain, and tissue remodeling [111].

### 4.3. α_2_-Adrenergic Agonists

α_2_-Adrenergic agonists such as dexmedetomidine and clonidine exhibit both analgesic and anti-inflammatory properties, making them attractive components of multimodal perioperative care. These agents exert their effects via presynaptic α_2_-adrenoceptors, inhibiting norepinephrine release and sympathetic outflow, while also attenuating NF-κB signaling and reducing pro-inflammatory cytokines such as TNF-α and IL-6 [112,113]. This dual mechanism enables neuroimmune modulation at the site of surgical injury, thus improving postoperative outcomes.

Dexmedetomidine, in particular, has shown broad therapeutic potential. Preclinical studies have demonstrated its capacity to suppress cytokine expression, reduce leukocyte infiltration, and preserve tissue architecture after surgical trauma [114,115]. Clinical trials across abdominal, orthopedic, and thoracic procedures further support its efficacy in reducing postoperative pain, opioid consumption, and systemic inflammation [116,117]. Despite these benefits, systemic administration is limited by dose-dependent side effects, including bradycardia, hypotension, and sedation, particularly in elderly or hemodynamically unstable patients [118,119].

To address these limitations, hydrogel-based delivery systems have been developed to localize and sustain the release of α_2_-agonists. In one approach, a mucoadhesive pH-sensitive hydrogel composed of carbopol and hydroxyethyl cellulose was formulated for sublingual dexmedetomidine administration. In rats, this system achieved high bioavailability (~89%) while reducing cardiovascular side effects compared with intravenous delivery [120]. Although not inflammation-responsive or wound-specific, its favorable pharmacokinetic profile and noninvasive format suggest a strong potential for adaptation in surgical contexts, particularly when combined with wound-responsive mechanisms.

Another platform integrated a clonidine-loaded thermoresponsive Pluronic F127 hydrogel with electrospun polycaprolactone nanofibers encapsulating ropivacaine. Upon perisciatic injection in rats, the composite system achieved prolonged sensory blockade (~32 h) with preserved motor function, outperforming free drug and single-component controls [121]. Although not designed to respond to inflammatory cues, its synergistic analgesic performance and sustained delivery support future translation into inflammation-adaptive hydrogel formats.

These examples highlight the potential of hydrogel-mediated α_2_-agonist delivery to enhance perioperative pain control and immune modulation while minimizing systemic toxicity. Incorporating pH-, ROS-, or protease-responsive features may further improve spatiotemporal precision, enabling context-specific targeting of neuroimmune pathways in surgical wound environments.

### 4.4. Biologics and Regenerative Factors

Biological agents, including cytokine inhibitors and regenerative growth factors, offer dual therapeutic benefits in perioperative care by suppressing inflammation and promoting tissue repair. However, their systemic administration is often limited by their rapid degradation, poor bioavailability, and unintended immune responses [122]. Hydrogel-based delivery systems can overcome these barriers by enabling localized sustained release. When engineered with pH-, ROS-, or protease-responsive components, these hydrogels synchronize drug release with the evolving inflammatory profiles of surgical wounds.

For cytokine inhibition, an MMP-responsive PEG hydrogel was developed by crosslinking an IL-1 receptor antagonist via cleavable peptide sequences. Upon MMP-1/2 activation, an IL-1 receptor antagonist was released to reduce neuroinflammation and protect the blood–brain barrier in a rat model of lipopolysaccharide-induced injury. Compared with uncoated or drug-free hydrogels, this platform improved neural interface outcomes [123]. Although originally developed for neurotrauma, the inflammation-adaptive mechanism of the system is directly applicable to perioperative scenarios where IL-1–mediated inflammation drives fibrosis and delayed healing.

To target TNF-α, infliximab was embedded in a modular scaffold composed of polydimethylsiloxane and porous PVA hydrogel. This platform provided stable antibody release for over 30 days in mice, significantly reducing local inflammation without compromising bioactivity [124]. Although not responsive to inflammatory cues, its architecture could support the future integration of protease-sensitive linkers or redox switches for adaptive cytokine control after surgery.

Another approach utilized nanovesicles displaying TNF receptor 1 incorporated into a thermosensitive Pluronic F127 hydrogel. In murine burn models, this system decreased TNF-α and IL-6 expression while accelerating epithelial regeneration relative to controls [125]. While release was thermally triggered, adaptation with inflammation-responsive matrices could extend its utility to surgical wounds that are at a high risk for persistent inflammation.

For regenerative applications, a bone morphogenetic protein (BMP)-2 delivery platform was created by combining mineral-coated microparticles with a chitosan–PEG hydrogel. In a rat calvarial defect model, this system provided sustained BMP-2 release for over 75 days and enhanced bone regeneration compared to free drug or blank gels [126]. Although not designed to respond to wound signals, the platform could benefit from responsiveness to pH or enzymatic cues that mark the transition from inflammation to repair.

In a vascular regeneration strategy, a VEGF-loaded PEG–maleimide hydrogel was crosslinked with MMP-cleavable VPM peptides and functionalized with the GFOGER integrin-binding motif. In a radial bone defect model, this design enhanced angiogenesis and bone healing compared to soluble VEGF or non-degradable controls [127]. Dual sensitivity to enzymatic and cellular cues reflects an advanced level of context-adaptive delivery that is relevant to perioperative tissue regeneration.

Collectively, these examples demonstrate the versatility of hydrogel-based systems for delivering biologics with spatial and temporal precision. Although most platforms remain preclinical and are not fully inflammation-responsive, their modular architectures and enzyme-cleavable elements provide a foundation for next-generation therapies that dynamically respond to the surgical wound environment. Future efforts should focus on integrating multiple endogenous triggers to optimize immune modulation, fibrosis control, and regenerative support during postoperative recovery.

### 4.5. Dual and Synergistic Payload Systems

Hydrogels that co-deliver analgesic and anti-inflammatory agents represent a powerful strategy for multimodal perioperative management. By combining local anesthetics with compounds such as α_2_-agonists, NSAIDs, or corticosteroids, these systems aim to achieve synergistic therapeutic effects while reducing systemic toxicity. Although many current designs are not fully inflammation-responsive, their modular architectures provide a foundation for integrating pH-, ROS-, or protease-sensitive elements to synchronize drug release with the evolving biochemical profiles of surgical wounds [128].

A notable example involves a dual-responsive hydrogel composed of GelMA and oxidized CS crosslinked through both pH- and ROS-sensitive linkers. This platform enabled inflammation-triggered release of curcumin from embedded micelles, resulting in enhanced epithelialization, increased collagen deposition, IL-10 upregulation, and suppression of IL-6 expression and bacterial burden in infected wound models [83]. While it lacked an anesthetic payload, the system demonstrates key design principles applicable to dual-agent formulations for perioperative care.

In another study, ropivacaine-loaded mesoporous silica microspheres and dexmedetomidine were co-encapsulated within a self-healing HA hydrogel. Although not responsive to inflammatory stimuli, the system provided prolonged sensory and motor blockade in a rat sciatic nerve model without neurotoxicity [129]. Its biocompatibility and sustained-release characteristics suggest its strong potential for adaptation into inflammation-sensitive formats tailored to postoperative pain trajectories.

A thermogelling poloxamer-based hydrogel co-loaded with bupivacaine and ketorolac demonstrated less than 2% burst release, and dual-drug delivery was sustained over 14 days in vitro [130]. While in vivo validation and responsiveness to inflammatory cues remain lacking, the injectability and mechanical stability of the formulation offer a practical base for future development into wound-adaptive delivery systems.

Finally, a compartmentalized core–shell hydrogel was engineered to sequentially release antimicrobial and regenerative factors. The outer shell provided antibiotic delivery for 5 days, followed by sustained BMP-2 and transforming growth factor-β1 release from the inner core over 2 weeks. In a dental wound model, this architecture enhanced bacterial clearance and odontogenic regeneration compared with single-agent or blank controls [131]. Although not triggered by endogenous inflammation, spatiotemporal layering of the platform provides a useful design template for coordinating complex perioperative therapies.

Collectively, these examples underscore the potential of dual and synergistic payload strategies to address the multifaceted needs of surgical wounds. Through co-delivery, sequential release, or structural compartmentalization, these systems enable more comprehensive perioperative modulation of pain, inflammation, and tissue healing. Further incorporation of inflammation-responsive elements will be critical for advancing these platforms toward clinical translation and precision-matched therapeutic delivery in dynamic postoperative environments.

Inflammation-responsive hydrogels provide versatile platforms for the localized and adaptive delivery of anti-inflammatory and analgesic agents, including NSAIDs, corticosteroids, α_2_-adrenergic agonists, and biologics, in alignment with the evolving biochemical landscape of surgical wounds. Dual-payload systems further extend this capability by integrating complementary mechanisms for pain control and immune modulation. Although most applications remain preclinical, these materials show strong potential to achieve sustained, context-sensitive perioperative therapy while minimizing systemic toxicity and supporting improved recovery outcomes. A concise summary of the payload classes, representative agents, and key delivery features is provided in Table 3.

## 5. Preclinical Applications in Perioperative Models

Inflammation-responsive hydrogels have emerged as promising tools for managing postoperative pain and wound healing through localized spatiotemporally controlled drug delivery. These systems are typically designed to respond to the key features of surgical inflammation, including elevated ROS, acidic pH, and upregulated protease activity, enabling therapeutic release to be synchronized with pathological cues at the wound site [18].

Although a few hydrogel platforms have demonstrated fully adaptive, inflammation-triggered release in surgical settings, an increasing number of preclinical studies have evaluated materials with partial or indirect responsiveness. These systems often outperform conventional delivery methods by improving drug retention, reducing systemic toxicity, and enhancing wound healing outcomes [132].

This section highlights the representative applications of inflammation-responsive hydrogels in preclinical models of soft tissue, orthopedic, thoracic, and abdominal surgery. We also compare these systems to alternative delivery strategies, such as bolus injection and microparticle depots, to illustrate the relative advantages of wound-adaptive hydrogels for analgesia, immune regulation, and tissue regeneration.

### 5.1. Soft Tissue Surgery

Soft tissue injuries, including incisional wounds, dental extractions, and abdominal wall repairs, represent frequent perioperative challenges characterized by acute inflammation, tissue disruption, and nociceptive pain [133]. Although relatively few hydrogel systems have been explicitly designed for surgical wound models, several preclinical platforms originally developed for trauma-induced or infected wounds exhibit mechanistic features and therapeutic outcomes that are well aligned with perioperative needs, particularly when incorporating responsiveness to ROS, acidic pH, or protease activity.

One representative system is a dual-responsive hydrogel composed of GelMA–CPBA and oxidized CS crosslinked through ROS-cleavable boronate esters and pH-sensitive Schiff bases. This matrix encapsulated curcumin-loaded micelles, enabling drug release under oxidative or acidic conditions. In a rat full-thickness infected wound model, the hydrogel promoted >95% closure by day 14, enhanced re-epithelialization, and induced M2 macrophage polarization relative to the free drug, micelle-only, and non-responsive hydrogel controls [83]. Although not tested in surgical incisions, its inflammation-adaptive delivery profile suggests potential for contaminated or ischemic wounds encountered perioperatively.

In another study, a conformal ROS-scavenging hydrogel was developed using a self-assembling oligopeptide functionalized with caffeic acid. When applied to deep scald wounds in rats, the system reduced TNF-α and IL-6 levels while enhancing angiogenesis, collagen organization, and re-epithelialization [134]. Its sprayable format and oxidative sensitivity make it a compelling candidate for irregular surgical wounds with a high ROS burden, although perioperative validation remains to be established.

Protease-responsive systems have also shown promise. An ultraviolet-crosslinked hydrogel composed of atelocollagen, retaining triple-helix integrity, was engineered to degrade in response to MMP-9. In a diabetic full-thickness wound model, it accelerated closure and epidermal regeneration compared to commercial dressings [44]. While designed for chronic wounds, its biomimetic architecture and enzyme-triggered degradation could translate well to surgical incisions or hernia repairs, where protease activity is elevated.

Altogether, these examples demonstrate that inflammation-responsive hydrogels, although often developed for non-surgical wound contexts, offer mechanistic advantages highly relevant to soft tissue surgery. Their capacity to modulate local inflammation, promote healing, and deliver drugs adaptively makes them promising candidates for future perioperative applications, pending rigorous validation in surgical models.

### 5.2. Orthopedic Surgery

Orthopedic procedures, such as fracture repair, tendon reconstruction, and joint preservation, are frequently associated with prolonged inflammation characterized by oxidative stress, MMP activation, and immune dysregulation. These factors not only impair structural healing but also contribute to persistent postoperative pain and functional limitations [135]. Inflammation-responsive hydrogels are being actively investigated as adaptive delivery systems that synchronize therapeutic release with injury-induced cues, including ROS, acidic pH, or protease activity, offering targeted and sustained modulation of the local microenvironment in musculoskeletal surgery.

A representative platform involves a hydrogel composed of dopamine-modified HA and CMCS crosslinked via ROS-labile boronate esters and imine bonds. This matrix encapsulated dimethyl fumarate and enabled ROS-triggered release in a rat calvarial defect model, resulting in enhanced bone regeneration and suppression of TNF-α, IL-6, and IL-1β expression [136]. Its injectability and immunomodulatory properties support its potential use in bone fractures and reconstructive surgeries involving elevated oxidative stress.

Enzyme-responsive delivery was demonstrated using a PEG hydrogel cross-linked with MMP-cleavable GPLGIAGQ peptides and functionalized with phosphatidylserine. In a calvarial bone defect model, this system facilitated phosphatidylserine release, increased bone volume, and promoted M2 macrophage polarization while downregulating inflammatory cytokines [75]. These features make it suitable for bone grafting or reconstruction of large defects, where immune resolution is critical.

For cartilage protection, an ROS-sensitive hydrogel incorporating magnetite nanoparticles was designed to deliver MMP-13–targeting siRNA. In a rat osteoarthritis model, this system reduced synovitis, inhibited cartilage degradation, and preserved type II collagen, outperforming controls [137]. Although developed for chronic inflammation, its inflammation-adaptive design may translate well to meniscal repair or cartilage-preserving surgeries requiring local gene modulation.

A particularly innovative design integrated both biochemical and biomechanical responsiveness. A piezoelectric hydrogel composed of PVA, cellulose nanofibers, and polydopamine-coated barium titanate nanoparticles generated electrical stimulation in response to joint motion. In vitro, it promoted M2 macrophage polarization and tenogenic differentiation, and in a rat rotator cuff repair model, it enhanced tendon–bone integration [138]. While early stage, this dual-responsive strategy offers promise for complex orthopedic repairs involving dynamic mechanical loading.

Finally, a dual pH- and ROS-responsive hydrogel composed of curcumin and cerium oxide nanoparticles encapsulated within a zeolitic imidazolate framework-8 was developed to prevent trauma-induced heterotopic ossification. In both murine and rat models, it suppressed aberrant osteogenesis by inhibiting the NF-κB and IL-17 pathways while preserving myogenic regeneration [139]. This strategy may be relevant for preventing ectopic bone formation following orthopedic trauma or surgical re-entry.

Collectively, these examples highlight the translational potential of inflammation-responsive hydrogels in orthopedic surgery. By aligning therapeutic release with the evolving local microenvironment, these systems offer precise immunomodulation and regenerative support for bone, cartilage, and tendon repair. Further validation in clinically relevant surgical models will be essential to refine their use in perioperative settings.

### 5.3. Thoracic and Abdominal Surgery

Thoracic and abdominal surgeries commonly provoke peri-neural and serosal inflammation, which contributes to postoperative adhesions, neuropathic pain, and organ dysfunction [140,141]. Inflammation-responsive hydrogels have been investigated in preclinical models of thoracotomy, laparotomy, and myocardial injury because of their capacity to localize and temporally control therapeutic release in response to oxidative stress, acidosis, or enzymatic activity.

In a representative thoracic application, a sprayable polypeptide hydrogel composed of poly-L-lysine and poly-L-glutamate was used as an anti-adhesive barrier in a rat thoracotomy model. The system reduced collagen deposition and pleural adhesion severity, and demonstrated intraoperative ease of use [142]. Although not designed with biological responsiveness, this baseline efficacy provides a foundation for the future integration of inflammation-adaptive triggers to enhance selectivity and therapeutic timing.

A cardiothoracic strategy employed an ROS-responsive hydrogel composed of quaternized CMCS and tannic acid. This matrix delivered two agents, a mitochondrial antioxidant (S1QEL1.1) and an anti-fibrotic compound (FT011), in a staged, inflammation-triggered manner. In a rat model of myocardial ischemia–reperfusion, the system preserved the mitochondrial ultrastructure, improved cardiac function, and reduced inflammation relative to blank or single-agent controls [143]. This example highlights the potential of coordinated stimulus-adaptive therapy in ischemic cardiac injury.

In a complementary approach, a multifunctional hydrogel integrated a photoadhesive PEG matrix with a conductive PEDOT:PSS–MXene mesh for secure vagus nerve electrode placement without sutures. In a myocardial infarction model, this system reduced perineural inflammation and prevented electrode displacement compared to commercial cuffs [144]. Although not inflammation-responsive, the stable bioelectronic interface enabled neuroimmune modulation, suggesting its promise for future perioperative neurotherapeutic applications.

In abdominal models, an ROS-degradable hydrogel formed by crosslinking succinylated HA–adipic dihydrazide and oxidized HA–epigallocatechin gallate released epigallocatechin gallate under oxidative conditions, reducing fibrosis and adhesion formation in a rat cecal abrasion model. The system promoted M2 macrophage polarization and suppressed TNF-α and inducible nitric oxide synthase expression, indicating dual immune and structural repair effects [145].

Another ROS-responsive hydrogel, composed of phenylboronic acid–functionalized HA and zwitterionic sulfobetaine polymers, enabled sustained delivery of chlorogenic acid. This platform decreased ROS levels, fibroblast adhesion, and postoperative adhesions in vivo [146]. Its dual action, through both pharmacological release and surface-mediated immune modulation, underscores the versatility of inflammation-adaptive hydrogels in abdominal wound environments.

Together, these studies illustrate the emerging role of inflammation-responsive hydrogels in thoracic and abdominal surgeries. By aligning drug delivery with peri-neural or serosal inflammation and, in some cases, integrating electronic or topographical modulation, these systems offer innovative strategies for reducing postoperative adhesions, controlling pain, and enhancing tissue regeneration.

### 5.4. Comparative Delivery Strategies in Preclinical Perioperative Models

Traditional drug delivery methods in the perioperative setting, such as systemic injections or bolus administration, often lead to fluctuating drug concentrations, poor tissue specificity, and inadequate control of localized inflammation. These shortcomings can delay wound healing and increase the risk of chronic pain or fibrosis [147]. In contrast, hydrogel systems designed to respond to surgery-induced stimuli, such as oxidative stress, acidosis, or altered perfusion, offer spatiotemporally controlled drug release that is better suited to the evolving demands of postoperative recovery [148].

One illustrative example comes from a rat sciatic nerve block model comparing bolus- and depot-based delivery. Lidocaine injected in saline produced approximately 2 h of analgesia, whereas a composite system combining lidocaine-loaded PLGA microspheres and a thermoresponsive poloxamer 407 hydrogel extended the block duration to 9 h [149]. Although not inflammation-responsive, the system formed an in situ depot with sustained release, highlighting the pharmacokinetic advantages of depot strategies over simple injections.

A more functionally adaptive system utilized staged release to optimize perioperative analgesia. Dexmedetomidine was encapsulated in biodegradable microspheres, while bupivacaine was dispersed within a hydrogel matrix. Dexmedetomidine was released first to induce local vasoconstriction, thereby reducing perfusion and prolonging the residence time of bupivacaine. In vivo, the system maintained analgesia for over 37 h [150]. Although not responsive to inflammatory biomarkers, this approach illustrates how hemodynamic cue–driven release timing can improve therapeutic outcomes.

In contrast, inflammation-responsive systems provide more precise control by aligning drug release with pathophysiological cues. A hydrogel composed of dopamine-modified HA and CMCS crosslinked via ROS-cleavable boronate esters enabled the targeted release of dimethyl fumarate under oxidative stress. In a rat cranial defect model, the platform suppressed TNF-α, IL-6, and IL-1β expression while promoting bone regeneration compared to the blank and non-responsive controls [136]. The on-demand release, self-healing behavior, and injectability of the system make it a strong candidate for orthopedic trauma care.

Similarly, a pH-sensitive hydrogel synthesized by grafting locust bean gum with poly(acrylamide-co-acrylic acid) was designed to release C-phycocyanin under alkaline wound conditions. In a full-thickness skin excision model, it accelerated wound closure, enhanced neovascularization, and reduced inflammatory cytokine levels relative to free drug and blank hydrogel controls [151]. Although not yet validated in surgical incisions, its pH-adaptive function suggests potential applicability in perioperative soft tissue repair.

Compared with current perioperative gold-standard approaches, such as systemic opioids, NSAIDs, single-shot regional blocks, and conventional anti-inflammatory or wound management regimens, these preclinical hydrogel systems have demonstrated meaningful quantitative gains in therapeutic window and, in some cases, improvements in side-effect profile. For example, lidocaine delivered as a saline bolus typically provides ~2 h of analgesia in rodent sciatic block models, whereas depot-based hydrogels extend this to ~9 h [149], and staged dexmedetomidine–bupivacaine hydrogels have maintained >37 h of analgesia in vivo [150]. Beyond pain control, inflammation-responsive and depot-forming hydrogels have improved other perioperative endpoints such as bacterial clearance, adhesion scores, and joint function compared with conventional therapies, as summarized in Table 4, which compiles representative quantitative outcomes across diverse surgical models. These systems have achieved multi-hour to multi-day analgesic coverage aligned with pathological cues, along with reductions in inflammatory cytokines relative to free-drug or blank controls [152]. While systemic pharmacokinetic data remain limited, localized hydrogel delivery is expected to lower peak plasma concentrations and thereby mitigate systemic adverse events commonly associated with opioids (e.g., nausea, sedation, respiratory depression) or NSAIDs (e.g., gastrointestinal and renal toxicity). Nonetheless, these comparative gains are currently supported primarily by preclinical studies, and validation in clinically relevant perioperative trials will be essential to confirm their translational value.

Together, these comparative studies highlight the functional advantages of inflammation-responsive hydrogels over conventional delivery strategies. By synchronizing drug release with local pathological cues, these systems offer enhanced tissue specificity, sustained pharmacodynamics, and reduced systemic toxicity. Compared to bolus injections or inert depots, stimulus-adaptive hydrogels provide superior control, therapeutic precision, and potential for tailored perioperative interventions.

Across a range of surgical models, including soft tissue, orthopedic, thoracic, and abdominal procedures, inflammation-responsive hydrogel systems have demonstrated significant potential for enhancing postoperative analgesia, immunomodulation, and tissue regeneration. By combining depot-like stability with pathophysiologically triggered release, these platforms offer advantages over traditional delivery methods in terms of duration of action, tissue specificity, and biocompatibility. Advancing stimulus-responsive chemistries, formulation strategies, and combinatory payload designs will be essential to accelerate clinical translation and optimize perioperative outcomes. A summary of the representative inflammation-responsive hydrogel platforms evaluated in preclinical perioperative models is provided in Table 5.

## 6. Translational Outlook and Future Directions: From Proof-of-Concept to Surgical Integration

Despite the growing interest in inflammation-responsive hydrogels for perioperative applications, their clinical translation remains in an early phase. As outlined in Section 1, Section 2, Section 3, Section 4 and Section 5, these platforms offer rationally designed stimulus-adaptive drug delivery strategies with the potential to achieve spatial precision, temporal control, and reduced systemic toxicity compared with conventional perioperative therapies [18,153].

However, several translational barriers remain to be overcome. Most preclinical evaluations have relied on static or chronic disease models, with limited validation in the physiologically dynamic and temporally complex environments of actual surgical procedures [16,18]. Furthermore, although pH-sensitive linkers, ROS-cleavable motifs, and enzyme-degradable matrices offer promising mechanisms of responsiveness, their integration into hydrogel systems that are clinically deployable and compliant with regulatory standards is rare [22,154]. As a result, a substantial gap remains between proof-of-concept efficacy and the development of functionally robust, surgically ready materials [24,26,155].

To address these limitations, this section critically examines the current maturity of preclinical models, design compatibility of inflammation-responsive hydrogels with standard surgical workflows, and challenges related to biocompatibility, immune reactivity, and trigger fidelity. It also explores emerging strategies, such as biosensor-coupled feedback systems, artificial intelligence (AI)-guided formulation platforms, and multi-input logic gating, which may facilitate the clinical adoption and surgical integration of these next-generation therapeutic materials.

### 6.1. Translational Gaps and Preclinical Readiness

Inflammation-responsive hydrogels have demonstrated promising therapeutic potential across various preclinical models by enabling drug release that dynamically aligns with the evolving wound microenvironment. However, their application in perioperative care remains largely exploratory. As discussed in Section 5.1, Section 5.2 and Section 5.3, most studies have employed simplified injury models, such as aseptic incisions or mechanical trauma, that only partially capture the complexity of surgical inflammation, which frequently involves electrocautery-induced damage, ischemia–reperfusion injury, and prolonged tissue manipulation.

A central limitation lies in the focus on general wound healing outcomes, such as re-epithelialization, collagen deposition, and angiogenesis, rather than endpoints directly relevant to perioperative inflammation and pain control [16,18]. Only a limited number of studies have incorporated standardized nociceptive assays, longitudinal cytokine profiling, or direct comparisons against clinical standards, such as systemic NSAIDs, long-acting anesthetic depots, or the U.S. Food and Drug Administration (FDA)-approved surgical dressings. This lack of rigorous benchmarking weakens the translational foundations of these technologies.

Nonetheless, select preclinical investigations have illustrated the functional advantages of inflammation-adaptive hydrogel design. For example, an ROS-scavenging hydrogel delivering apelin-13 promoted M2 macrophage polarization and improved locomotor recovery in a rat spinal cord injury model [156], while an MMP-cleavable PEG matrix facilitated synchronized degradation and immune-mediated bone regeneration in murine cranial defects [157]. Although these models fall outside the conventional surgical domains, they highlight the mechanistic value of responsiveness to inflammatory cues.

Despite these encouraging findings, most studies are constrained by limited follow-up durations (typically ≤7 days), restricting the evaluation of long-term therapeutic durability, late-phase inflammatory remodeling, and functional recovery. Surgical fidelity is often limited, with models failing to capture the full spectrum of perioperative injury factors and tissue responses observed in clinical settings. Methodological weaknesses are also common: many investigations rely on blank or untreated comparators rather than established perioperative standards, use small sample sizes that limit statistical power, and omit randomization or blinded outcome assessment. Furthermore, short observation windows and the absence of standardized, clinically relevant endpoints, such as validated nociceptive assays, longitudinal cytokine profiling, or pharmacokinetic comparisons, reduce the strength of the evidence base. Collectively, these limitations undermine statistical robustness, increase susceptibility to bias, and weaken both the translational validity and regulatory acceptability of current findings [23,158].

Bridging these gaps will require a strategic shift from exploratory efficacy studies to perioperative evaluation pipelines grounded in clinically meaningful endpoints. Future research should incorporate validated pain metrics, dynamic immune profiling, retention and degradation kinetics, and comparative performance with approved perioperative therapies. Establishing a consensus set of benchmark parameters would further enable reproducible, cross-platform evaluation of inflammation-responsive hydrogels. Key recommended metrics include:

1. Trigger fidelity—specificity and sensitivity to pathological pH, ROS, or protease thresholds.
2. Release kinetics—under both simulated and in vivo perioperative inflammatory conditions.
3. Mechanical stability and degradation—including timeline and identification of degradation byproducts.
4. Inflammatory biomarker modulation—e.g., TNF-α, IL-6 at defined postoperative timepoints.
5. Therapeutic window—duration of analgesic or anti-inflammatory effects compared with current clinical standards.
6. Functional endpoints—standardized nociceptive assays, mobility scores, or tissue regeneration quality.
7. Safety—assessment of local tissue compatibility and systemic toxicity.

In parallel, the development of standardized surgical inflammation models incorporating clinically relevant injury mechanisms, such as electrocautery, ischemia–reperfusion, and multi-tissue handling, will be essential for robustly assessing hydrogel efficacy and safety. Such models, coupled with harmonized endpoint criteria, would improve reproducibility, facilitate head-to-head comparisons, and better align preclinical testing with the complex inflammatory milieu of real surgical settings.

### 6.2. Regulatory Compatibility and Surgical Integration

To transition from preclinical innovation to clinical adoption, inflammation-responsive hydrogels must align with existing regulatory frameworks and integrate seamlessly into surgical workflows. Regulatory authorities, such as the U.S. FDA and the European Medicines Agency typically categorize these platforms, particularly those combining structural matrices with bioactive agents, as combination products. As such, they are subject to dual regulatory oversight for both device and pharmaceutical components [25]. The lead regulatory pathway is determined by the product’s primary mode of action, whether drug, biologic, or device, and mandates comprehensive evaluation of safety, efficacy, and manufacturing quality in accordance with guidelines from the International Council for Harmonization and the International Organization for Standardization (ISO) [26].

Encouragingly, many inflammation-responsive hydrogel platforms are based on clinically established polymers such as PEG, HA, and chitosan, materials already approved for drug depots, surgical sealants, and ophthalmic products [21,159,160]. This regulatory familiarity may expedite approval pathways, provided that the integration of responsive chemistries such as ROS-cleavable boronate esters, pH-sensitive imines, or MMP-degradable peptides does not introduce novel toxicological liabilities or stability issues. These responsive elements must undergo rigorous validation not only for trigger fidelity and release kinetics, but also for long-term biocompatibility, degradation behavior, and potential byproducts under physiological conditions [161].

Equally critical is ensuring compatibility with the surgical workflow. Clinically deployable hydrogel systems must adopt formats amenable to intraoperative use, including injectable sols, thermogelling liquids, sprayable coatings, or brush-on precursors that polymerize or solidify in situ without requiring sutures or complex instrumentation [162]. Material properties such as gelation time, viscosity, mechanical compliance, adhesiveness, and optical clarity must be tailored to fit specific procedural contexts, particularly in minimally invasive settings such as laparoscopy, thoracoscopy, or microsurgery [96]. Matching crosslinking profiles and working times to surgical tempos can further enhance integration into routine operative care [161].

Despite substantial laboratory progress, relatively few inflammation-responsive hydrogels have successfully navigated critical translational milestones, including Good Manufacturing Practice (GMP)-compliant scale-up, ISO 10993 biocompatibility testing, and human factor evaluation in simulated surgical environments [23,24]. These steps are essential not only for regulatory approval but also for ensuring surgeon confidence, device familiarity, and operational adoption by perioperative teams.

Ultimately, successful clinical translation will require a balance between therapeutic efficacy, material sophistication, and practical usability. Hydrogel platforms that are modular, compositionally stable, and compatible with existing excipients, devices, and surgical workflows will be best positioned to advance from the bench to the bedside. Considering current regulatory requirements, manufacturing constraints, and the need for validation in clinically relevant surgical models, the earliest realistic timeline for initiating first-in-human trials of inflammation-responsive hydrogels in perioperative settings is likely within the next decade. This projection reflects the substantial time required to complete reproducible safety and efficacy studies under Good Laboratory Practice conditions, address trigger specificity, and engage regulatory agencies early to facilitate translational readiness and approval pathways.

### 6.3. Key Barriers: Biocompatibility and Responsiveness

Although inflammation-responsive hydrogels offer a compelling strategy for targeted and adaptive drug delivery, their clinical translation is hindered by persistent technical and biological challenges [12]. The chief among these is the difficulty of achieving precise, reproducible responsiveness to pathological stimuli, such as elevated ROS, MMP activity, or acidic pH, without compromising biocompatibility or inducing off-target effects in non-inflamed tissues [163].

A central obstacle lies in tuning the degradation kinetics and drug release profiles to align with the temporal dynamics of postoperative inflammation. Responsive linkers must degrade within clinically relevant windows, typically spanning several hours to a few days, while avoiding premature or delayed activation. Overly sensitive materials risk responding to baseline physiological ROS or enzyme levels, whereas under-responsive systems may fail to deliver therapy during critical inflammatory phases. Calibrating these thresholds in vivo remains a significant challenge, particularly given the inter-tissue and inter-patient variability in inflammatory profiles. Mechanistically informed designs that leverage real-time profiling of wound biomarkers may enhance trigger specificity and improve therapeutic precision [164].

A second major challenge concerns the complex immunological interactions of responsive hydrogel systems. While certain platforms support M2 macrophage polarization and contribute to inflammation resolution, others may provoke unintended immune activation owing to their degradation byproducts, nanoscale architecture, or crosslinking chemistries [165,166]. The inherent plasticity of immune responses, especially macrophage phenotype switching and cytokine feedback dynamics, can significantly alter hydrogel behavior in vivo, introducing variability and risk of adverse tissue remodeling [167].

Moreover, many responsive systems lack sufficient selectivity for pathological inflammation. For instance, ROS levels can be elevated due to ischemia, hypoxia, or basal metabolic activity, independent of overt inflammation, and MMPs play roles in both physiological and pathological tissue remodeling. This lack of specificity can result in off-target activation, uncoordinated drug release, and premature depletion of therapeutic payloads [168]. To address this challenge and discriminate between physiological and pathological levels of inflammatory markers, hydrogel systems can incorporate calibrated activation thresholds and buffer capacity tuning to restrict responsiveness to pathological ranges. Logic-gated multi-stimuli designs (e.g., AND-gated systems requiring concurrent pathological cues) and feedback-controlled or adaptively crosslinked networks can further refine trigger specificity, aligning drug release with the evolving wound environment [90,91]. Validation against clinically relevant biomarker ranges in perioperative surgical models will be essential to ensure trigger fidelity and translational robustness.

Inter-individual variability adds an additional layer of complexity. Patient-specific factors such as age, comorbidities (e.g., diabetes, cardiovascular disease), immune status, and concurrent medications influence the perioperative inflammatory milieu and, consequently, the performance of responsive hydrogels. Addressing this heterogeneity may require modular or customizable formulations tailored to individual wound profiles. For example, older patients or those with metabolic disorders may exhibit prolonged low-grade inflammation, impaired angiogenesis, or altered cytokine trajectories, which could necessitate hydrogels with extended or staged release kinetics to sustain therapeutic coverage over an extended healing period [169,170]. Conversely, immunocompromised patients or those at high risk of infection may benefit from lower activation thresholds, accelerated drug deployment during the early inflammatory phase, and payloads optimized for antimicrobial and pro-regenerative balance [171]. Patients with heightened baseline oxidative stress, such as those with cardiovascular disease or chronic smoking history, may require ROS-triggered systems with tighter threshold calibration or logic-gated designs to avoid premature payload depletion [172,173]. Integration with biosensors, feedback-controlled delivery systems, or AI-assisted wound profiling can further enable personalized trigger calibration, payload selection, and dosing schedules [174]. By incorporating these patient-specific considerations at the design stage, inflammation-responsive hydrogels can be more effectively matched to the clinical and biological context of each surgical case, improving both safety and therapeutic efficacy.

From a manufacturing and quality control standpoint, ensuring reproducible stimulus–response behavior across production batches remains a critical translational hurdle. Variability in raw material purity, crosslinking efficiency, and incorporation of responsive linkers can shift activation thresholds and alter release kinetics, undermining trigger fidelity. These risks are amplified for chemistries such as ROS-cleavable boronate esters, pH-sensitive imines, or MMP-degradable peptides, which may be sensitive to moisture, light, or sterilization [66,175]. To address this, production workflows should integrate GMP principles early, with precise control over polymer composition, crosslink density, and functional group integration. Quality control protocols should extend beyond conventional physicochemical assays to include trigger-specific performance testing under standardized simulated perioperative conditions, ensuring responsiveness within validated pathological ranges. Stability studies to confirm preserved functionality after storage and sterilization, along with high-throughput or automated in-line monitoring to detect batch deviations, will be essential to maintain clinical-grade reliability. Importantly, scalability of manufacturing must also be validated, as responsive behavior observed at bench scale may not translate directly to large-batch production, necessitating robust process controls and reproducibility benchmarks at every scale-up stage [176,177].

A further safety consideration unique to inflammation-responsive hydrogels is the potential for distinct degradation products and immune responses that are not typically encountered with conventional non-responsive depots. These systems often rely on stimulus-cleavable linkers such as thioketals, boronate esters, or protease-sensitive peptides. The degradation of these chemistries can yield byproducts with distinct chemical reactivity, solubility, or bioactivity, which may influence local tissue compatibility and systemic exposure [178]. While many responsive platforms are designed for biocompatible degradation into inert fragments, incomplete cleavage or secondary oxidation or reduction reactions can produce reactive intermediates capable of amplifying local inflammation or inducing oxidative stress [179]. The spatiotemporal pattern of activation, which is often concentrated within inflamed tissues, can also trigger acute bursts of drug release and transiently high local concentrations of degradation products [180]. These scenarios are less common in conventional sustained-release systems. Immune recognition of novel crosslinkers or their breakdown products may elicit hypersensitivity, complement activation, or unintended macrophage polarization shifts [181]. Accordingly, preclinical evaluation should extend beyond standard cytotoxicity and ISO 10993 biocompatibility testing to include immunoprofiling under relevant pathological conditions, longitudinal monitoring of degradation products, and systemic biodistribution analysis [182]. Such safety characterization will be essential to ensure that responsive platforms maximize their therapeutic advantage while safeguarding against new risks.

Overall, advancing inflammation-responsive hydrogels toward clinical reliability will necessitate rigorous optimization of trigger fidelity, immune compatibility, and temporal control. Mechanistically guided, clinically validated designs will be essential to bridge the gap between laboratory innovation and consistent performance in surgical settings.

### 6.4. Next-Generation Strategies for Precision Translation

To address the challenges outlined in the previous sections, next-generation hydrogel platforms are embracing multifunctional and intelligent design principles aimed at enhancing translational precision. These emerging strategies integrate advances in materials science, bioengineering, and computational modeling to improve stimulus specificity, surgical usability, and therapeutic personalization [183].

One promising direction is the development of modular hydrogel architectures that allow interchangeable components, such as stimulus-responsive linkers, drug reservoirs, and mechanical scaffolds, to be tailored to specific surgical applications. This design flexibility facilitates the rapid optimization of release kinetics, degradation rates, and immune interactions [184]. For example, PEG- or chitosan-based scaffolds can be selectively functionalized with ROS-cleavable or enzyme-sensitive moieties, depending on the dominant inflammatory pathway in a given tissue [12].

Three-dimensional printing and biofabrication techniques further enable precise spatial control over hydrogel geometry, porosity, and drug distribution. Printed hydrogel dressings or implants can be engineered to conform to complex anatomical sites, incorporate stratified drug layers, or localize bioactive cues that guide differential tissue regeneration. These strategies are particularly relevant for reconstructive and vascular surgeries, where anatomical precision and biomechanical compatibility are critical [185].

Transformative frontiers involve integration of biosensors into hydrogel matrices. These “smart” systems embed chemical or electronic sensors capable of detecting ROS, pH, or MMP levels in real time, enabling closed-loop feedback-controlled drug release. Prototype platforms have demonstrated their ability to detect cytokine surges and electronically trigger drug diffusion. Although not yet validated in surgical settings, such technologies offer significant potential for dynamic perioperative modulation [186].

In parallel, AI approaches, including computational modeling and machine learning are increasingly employed to accelerate hydrogel design. Algorithms trained on polymer structures, stimulus–response kinetics, and biological outcomes have been used to predict key parameters such as mechanical strength, degradation rates, and drug release profiles, thereby reducing the number of experimental iterations required for formulation development [187]. In one recent demonstration, a model was trained on a dataset of 180 bioinspired hydrogel formulations, incorporating composition, crosslinking chemistry, and adhesive performance, and was able to identify new compositions with adhesive strengths exceeding 1 MPa, which were subsequently validated experimentally [188]. Similar strategies could be applied to inflammation-responsive hydrogel research by developing datasets that capture surgical wound dynamics, including cytokine profiles, ROS trajectories, and pH changes, to inform the design of personalized and responsive hydrogel architectures. Coupling AI-guided prediction with high-throughput screening, automated synthesis, and real-time characterization may further bridge the gap between bench-scale innovation and clinically scalable platforms. Despite challenges such as limited domain-specific datasets and the difficulty of modeling complex in vivo environments, AI-guided formulation has strong potential to enhance the adaptability, precision, and translational readiness of perioperative hydrogel therapeutics.

Another promising avenue involves logic-gated and multi-stimuli-responsive hydrogels. These systems require the concurrent presence of two or more pathological signals, such as low pH and elevated MMPs, to trigger drug release. Inspired by synthetic biology, these AND-gated constructs enhance delivery precision by reducing premature activation and improving spatial selectivity. Although initial demonstrations have focused on tumor and chronic wound models, adaptation to surgical contexts is actively underway [189].

While not yet represented in standardized translational frameworks, extending the design of inflammation-responsive hydrogels to encompass microbiological control represents a strategically significant translational direction. Surgical site infections remain a major source of postoperative morbidity, with bacterial adhesion and biofilm formation posing persistent challenges to implanted biomaterials [190]. Future inflammation-responsive platforms could integrate antimicrobial payloads, quorum-sensing inhibitors, or surface modifications that resist bacterial colonization, thereby addressing both inflammatory and infectious components of the wound microenvironment [191,192]. Such dual-function systems would be particularly valuable in high-risk surgical settings, enabling synchronized modulation of immune responses and microbial burden within clinically relevant perioperative windows.

Together, these next-generation strategies represent a paradigm shift in hydrogel development from passive carriers to intelligent, adaptive therapeutic systems. Rather than incremental advances, the field is converging toward integrated platforms that combine biosensing, logic-based responsiveness, and personalized programmability. These technologies constitute a critical step toward realizing the clinical potential of inflammation-responsive hydrogels in perioperative care.

While certain inflammation-responsive hydrogel systems, particularly those with broad-spectrum anti-inflammatory or analgesic payloads, could function as standalone interventions in select surgical scenarios, their greatest translational potential is likely within multimodal perioperative care pathways [193]. Integration with systemic pharmacotherapy, regional anesthesia techniques, antimicrobial prophylaxis, and advanced wound management can provide complementary mechanisms of action, enhancing both efficacy and safety. Such combinatorial strategies could also reduce the dosing burden of individual agents, minimize systemic toxicity, and address multiple pathophysiological processes concurrently. Designing hydrogel platforms with compatibility for co-administration or sequential application alongside existing perioperative protocols will therefore be a critical step toward their routine clinical adoption.

A critical next step for the field will be a preclinical study designed to address the most important translational question: can inflammation-responsive hydrogels selectively activate in the presence of pathological inflammatory cues while remaining inert under physiological conditions, across diverse surgical models and patient-relevant comorbidity profiles? This question is central to demonstrating both the safety and efficacy required for clinical adoption. Such a study should include side-by-side comparisons with non-responsive depots and bolus delivery of the same payload, enabling quantitative assessment of spatial targeting, dosing precision, and safety. Evaluation should extend beyond conventional efficacy endpoints to incorporate longitudinal biomarker tracking, degradation product characterization, and immune profiling, ensuring that activation thresholds and therapeutic windows are validated against clinically relevant perioperative ranges. Addressing this question would generate decisive translational evidence and provide a rational foundation for the design of first-in-human trials.

In conclusion, while inflammation-responsive hydrogels are conceptually aligned with the physiological demands of surgical recovery, their successful deployment hinges on overcoming several translational bottlenecks. As outlined in Section 6, current preclinical models offer valuable mechanistic insights but often fall short in procedural realism and regulatory alignment. Encouragingly, recent innovations, including biosensor integration, AI-assisted formulation, and workflow-compatible delivery formats, represent a viable path forward. Bridging innovative material designs with clinical pragmatism will be essential to translate these smart biomaterials into effective, next-generation perioperative therapeutics. Table 6 summarizes the key translational challenges and corresponding strategies that can facilitate the integration of inflammation-responsive hydrogels into perioperative clinical practice.

## 7. Conclusions

Inflammation-responsive hydrogels represent a transformative paradigm in perioperative therapy, offering localized and spatiotemporally controlled delivery of analgesic and anti-inflammatory agents in direct response to dynamic wound cues. As outlined in this review, these systems, engineered to respond to stimuli such as ROS, acidic pH, and matrix-degrading enzymes, address the key limitations of conventional approaches, including systemic toxicity, inconsistent drug exposure, and poor tissue targeting. A growing body of preclinical evidence supports their feasibility across diverse surgical models, from soft tissue repair to orthopedic and thoracoabdominal procedures.

Despite these advances, its clinical translation remains in its infancy. Most platforms have not yet been evaluated in realistic surgical environments, and current preclinical studies often prioritize structural endpoints, such as wound closure and histology, over functional outcomes related to pain control, immune modulation, and pharmacokinetics. The incorporation of responsive mechanisms into clinically viable and GMP-compatible hydrogel systems poses significant technical and regulatory challenges. Additional barriers include interpatient variability in inflammatory signatures, difficulty in fine-tuning release kinetics, potential immune responses to degradation products, and a lack of validated real-time biomarkers for trigger fidelity.

Looking ahead, the successful implementation of inflammation-responsive hydrogels will depend on bridging innovative material science with a clinically grounded design. Future platforms must integrate modular architectures, biosensor-mediated feedback, logic-based activation schemes, and AI-guided formulations to accommodate the heterogeneity of surgical wounds and patient-specific immune responses. Realizing this vision will require sustained interdisciplinary collaboration among biomaterial scientists, surgeons, immunologists, and regulatory experts.

If these challenges can be overcome, inflammation-responsive hydrogels hold immense potential to transform perioperative care, enabling therapeutics that are not only responsive and intelligent, but also safe, scalable, and seamlessly integrated into surgical workflows. By aligning drug delivery with the biology of wound healing, these systems may not only redefine the standard of care in surgical recovery but also usher in a new era of precision biomaterials powered by dynamic, biomarker-responsive designs.

## Figures and Tables

**Table 1:** Key phases of perioperative inflammation.

Inflammatory Phase	Key Triggers	Design Implications
Acute (0–72 h)	ROS, low pH, NE, MMPs, IL-1β	Burst or early-phase release via ROS-, pH-, or enzyme-responsive linkers
Resolution (>72 h)	MMP-2/9, IL-10	Sustained release of regenerative agents; ECM-mimetic or degradable scaffolds
Spatial variation	Local hypoxia, edema, cytokine gradients	Injectable, conformal, or multi-stimuli systems for site-specific delivery

ECM, extracellular matrix; IL, interleukin; MMP, matrix metalloproteinase; NE, neutrophil elastase; pH, potential of hydrogen; ROS, reactive oxygen species.

**Table 2:** Classes of Inflammation-Responsive Hydrogels.

Hydrogel Class	Primary Trigger	Mechanism of Response	Representative Design Strategy
pH-responsive	Local acidosis (pH 6.0–6.8)	Hydrolytic cleavage or ionic swelling	Orthoester, acetal, imine linkers; ionizable PAA
ROS-responsive	H_2_O_2_, O_2_^−^, OH^−^ radical	Oxidative cleavage or crosslink disruption	Thioketal, boronic ester, diselenide bonds
Enzyme-responsive	MMP-2/9, NE	Protease-mediated peptide cleavage	GPLGIAGQ or AAPV peptide crosslinkers
Multi-stimuli/feedback	ROS + pH, MMP + pH, cytokines	Boolean logic (AND/OR), biosensor-mediated release	Dual-labile networks, aptamer-functionalized matrices, redox-switchable gels

H_2_O_2_, hydrogen peroxide; MMP, matrix metalloproteinase; NE, neutrophil elastase; O_2_^−^, superoxide anion; OH^−^, hydroxyl radical; PAA, poly(acrylic acid); ROS, reactive oxygen species.

**Table 3:** Representative therapeutic payloads delivered via inflammation-responsive hydrogels in perioperative settings.

Payload Class	Key Examples	Delivery Features
NSAIDs	Diclofenac, Ketoprofen, Celecoxib	pH-/ROS-responsive; improved local efficacy
Corticosteroids	Dexamethasone, Triamcinolone	Sustained release; enzyme-triggered systems
α_2_-Adrenergic Agonists	Dexmedetomidine, Clonidine	Mucoadhesive or thermosensitive hydrogels
Biologics/Regenerative Factors	IL-1Ra, TNF-α blockers, BMP-2, VEGF	Cytokine/ECM-targeted; protease-responsive
Dual-Payload Systems	Ropivacaine + Dexmedetomidine or Ketorolac	Synergistic release; compartmental structures

BMP, bone morphogenetic protein; ECM, extracellular matrix; IL-1Ra, interleukin-1 receptor antagonist; NSAIDs, non-steroidal anti-inflammatory drugs; ROS, reactive oxygen species; TNF-α, tumor necrosis factor-alpha; VEGF, vascular endothelial growth factor.

**Table 4:** Quantitative comparison of inflammation-responsive hydrogel systems and conventional comparators in preclinical perioperative models.

Hydrogel Example	Trigger Type	Payload	Model	Comparator	Key Quantitative Outcomes	Ref.
Dual-responsive gelatin–PVA	ROS + pH	Vancomycin–Ag NCs + nimesulide	Rat diabetic infected wound	Commercial dressing	Wound closure ↑ (92% vs. 74%, *p* < 0.01), TNF-α ↓ (~45%, *p* < 0.01), Bacterial clearance ↑ (96% vs. 62%, *p* < 0.001)	[69]
MMP-cleavable tetra-PEG	Enzyme (MMP-2/9)	Phosphatidylserine	Rat calvarial defect	Non-responsive hydrogel	Bone regeneration ↑ (78% vs. 54%, *p* < 0.05), IL-1β ↓ (~40%, *p* < 0.05), M2/M1 ratio ↑ (~3.2-fold, *p* < 0.01)	[75]
MMP-13-responsive HAMA microsphere	Enzyme (MMP-13)	Celecoxib	Rat OA model (ACLT + MMx)	Non-responsive hydrogel	OARSI score ↓ (~75%, *p* < 0.05), Joint space width maintained, Col2 preserved	[100]
MMP-cleavable PEG	Enzyme (MMP-1/2)	IL-1Ra	Rat cortical LPS-induced neuroinflammation model	Uncoated implant	IgG leakage ↓ to sham level (*p* < 0.05), Neuronal survival ↑ (*p* < 0.05)	[123]
ROS-degradable HA–EGCG	ROS	EGCG	Rat cecum–sidewall abrasion adhesion model	Commercial HA hydrogel	Adhesion score ↓ (1.2 vs. 3.8, *p* < 0.05), Fibrosis markers ↓, M2 macrophages ↑	[145]
ROS-responsive HA–PBA/ADH + PVA–SB–CHO	ROS	Chlorogenic acid	Rat cecum–sidewall abrasion adhesion model	Commercial HA hydrogel	Adhesion score ↓ (1.0–1.125 vs. 3.5+, *p* < 0.0001), 20% adhesion-free	[146]

ACLT, anterior cruciate ligament transection; ADH, adipic dihydrazide; Ag NCs, silver nanoclusters; CHO, aldehyde; EGCG, epigallocatechin gallate; HA, hyaluronic acid; HAMA, methacrylated hyaluronic acid; IL-1β, interleukin-1 beta; IL-1Ra, interleukin-1 receptor antagonist; LPS, lipopolysaccharide; MMx, medial meniscectomy; MMP, matrix metalloproteinase; OA, osteoarthritis; OARSI, Osteoarthritis Research Society International; PBA, phenylboronic acid; PEG, polyethylene glycol; PVA, polyvinyl alcohol; ROS, reactive oxygen species; SB, salicylidene benzoyl hydrazone; TNF-α, tumor necrosis factor-alpha.

**Table 5:** Summary of inflammation-responsive hydrogel applications in preclinical perioperative models.

Surgical Model	Responsive Cue	Main Outcome
Incisional wound	ROS, pH	Reduced inflammation, faster healing
Burn injury	ROS	Lower cytokines, improved repair
Bone fracture	MMP, pH	Enhanced bone regeneration
Joint surgery	Enzyme	Cartilage protection
VATS/laparotomy	pH, ROS	Nerve protection, reduced pain

MMP, matrix metalloproteinase; ROS, reactive oxygen species; VATS, video-assisted thoracoscopic surgery.

**Table 6:** Key challenges and strategies for clinical translation of inflammation-responsive hydrogels.

Challenge Area	Current Limitation	Emerging Strategy
Preclinical Models	Non-surgical, short-term, poor benchmarking	Standardized surgical inflammation models incorporating clinically relevant injury mechanisms (e.g., electrocautery, ischemia–reperfusion, multi-tissue handling) and harmonized benchmark endpoints (trigger fidelity, release kinetics, mechanical stability, biomarker modulation, therapeutic window, functional recovery, safety)
Responsiveness Fidelity	Off-target release, low specificity	Logic-gated or biosensor-integrated systems
Biocompatibility	Immunogenicity, unstable degradation byproducts	Modular, clinically validated chemistries
Workflow Integration	Complex delivery, poor fit with surgical pace	Injectable, sprayable, or thermogelling formats
Regulatory Translation	Combination product hurdles, GMP gaps	Use of ISO-approved polymers, early alignment

GMP, Good Manufacturing Practice; ISO, International Organization for Standardization.

## Data Availability

No new data were created or analyzed in this study. Data sharing is not applicable to this article.

## Abbreviations

The following abbreviations are used in this manuscript:

NSAID	Non-steroidal anti-inflammatory drug
ROS	Reactive oxygen species
IL	Interleukin
TNF-α	Tumor necrosis factor-α
NE	Neutrophil elastase
MMP	Matrix metalloproteinase
ECM	Extracellular matrix
COX	Cyclooxygenase
PAA	Poly(acrylic acid)
PEG	Polyethylene glycol
PVA	Poly(vinyl alcohol)
PLGA	Poly(lactic-co-glycolic acid)
GelMA	Gelatin methacryloyl
VEGF	Vascular endothelial growth factor
OCP	Octacalcium phosphate
CPBA	Carboxyphenylboronic acid
CS	Chondroitin sulfate
CMCS	Carboxymethyl chitosan
HA	Hyaluronic Acid
BMP	Bone morphogenetic protein
AI	Artificial intelligence
FDA	Food and Drug Administration
ISO	International Organization for Standardization
GMP	Good Manufacturing Practice
